# Radiotherapy improves serum fatty acids and lipid profile in breast cancer

**DOI:** 10.1186/s12944-017-0481-y

**Published:** 2017-05-18

**Authors:** Sana Shaikh, Naseem Aslam Channa, Farha Naz Talpur, Muhammad Younis, Naila Tabassum

**Affiliations:** 1 Institute of Biochemistry, University of Sindh, Sindh, Jamshoro, Pakistan; 2 National Centre of Excellence in Analytical Chemistry, University of Sindh, Jamshoro, 76080 Pakistan; 3 The key Laboratory of Development Genes and Human Diseases, Institute of Life Sciences, Southeast University, Sipailou 2, Nanjing, 210096 China

**Keywords:** Radiotherapy, Breast cancer, Lipids, Fatty acids, Gas chromatography

## Abstract

**Background:**

Breast cancer is a disease with diverse clinical symptoms, molecular profiles, and its nature to response its therapeutic treatments. Radiotherapy (RT), along with surgery and chemotherapy is a part of treatment in breast cancer. The aim of present study was to investigate pre and post treatment effects of radiotherapy in serum fatty acids and its lipids profile in patients with breast cancer.

**Methods:**

In this comparative as well as follow up study, Serum fatty acids were performed by gas chromatography to investigate fatty acids and Microlab for analysis of lipid profile.

**Results:**

Among serum free and total fatty acids the major saturated fatty acids (SFAs) in serum lipids of breast cancer patients (pre and post treated) were stearic acid (18:0) and palmitic acid (16:0). These fatty acids contributed about 35-50% of total fatty acids. The decreased concentrations of linoleic acid (C18:2) and arachidonic acid (C20:4) with a lower ratio of C18:2/C18:1 was found in pretreated breast cancer patients as compared to controls. The n-3/n-6 ratio of breast cancer patients was decreased before treatment but it was 35% increased after treatment. In addition, plasma activity of D6 desaturase was increased in the breast cancer patients, while the activity of D5 desaturase was decreased. Increased levels of SFAs, monounsaturated fatty acids (MUFAs) and decreased polyunsaturated fatty acids (PUFAs) levels in breast cancer patients (pre and post treated) as compared to controls. Serum total cholesterol (TC) (224.4 mg/dL) and low density lipoprotein cholesterol (LDL-C) (142.9 mg/dL) were significantly increased in pretreated breast cancer patients but after the radiotherapy treatment, the TC (150.2 mg/dL) and LDL-C (89.8 mg/dL) were decreased.

**Conclusion:**

It seems that RT would have played a potential role in the treatment of BC. After RT the serum levels of PUFAs, TC, and LDL-C are improved. Our study reinforces the important role of RT in the management of BC.

The level of PUFAs, TC, and LDL-C can be used as the biomarkers for early diagnosis in individuals with risk of breast cancer.

**Electronic supplementary material:**

The online version of this article (doi:10.1186/s12944-017-0481-y) contains supplementary material, which is available to authorized users.

## Background

Breast cancer (BC) is a disease with diverse molecular profiles, clinical behavior and response to therapy [1]. Radiotherapy (RT) is being used to treat cancer patients, since very long time. Now a day, more than half of all the cancer patients receive RT at one point during their treatment. RT after surgery plays an important role in the treatment of early and advance stage in BC. Recurrence is the main problem in the management of BC, but RT lowers the local recurrence, improves survival rates and controls the growth of cancerous cells in BC [2]. Modern RT techniques have been developed for the improvement of temporary tolerability along with a reduction in tissue damage in BC patients [3].

Dietary lipids are found to have an association of BC recurrence and survival of cancerous cells. Lipids as the part of cell membranes signal molecules and energy substrates play a vital role in BC. Previous studies reported a correlation of fat and suggested that dietary fat plays an important role in the incidence of BC, in an animal model [4]. Dietary fat is directly associated with BC as the high fat intake is composed of fatty acids which have distinctive biophysical and chemical properties that can influence on BC disease and normal health [5, 6]. Saturated fatty acids (SFAs) and monounsaturated fatty acids (MUFAs) are reported with the possible association with increased risk of BC in rodents [7] and humans [8]. It is proved that BC synthesizes endogenously 95% of FAs for nutritional lipid supply. SFAs and MUFAs positively associated are reported with the risk of BC [9]. Polyunsaturated fatty acids (PUFAs) are an important part of the diet, sufficient amount of PUFAs in the diet is essential because they are structural components of cell membrane and play an important role in cell signaling, metabolism, regulation of gene expression and inflammation [10]. Some animal studies found that n-6 PUFAs are associated with tumor enhancing effects while n-3 PUFAs shows protective effects [7]. The n-3 PUFAs may be associated with several mechanisms that counteract carcinogenic processes [11, 12]. Another study found that saturated fat increased the risk of BC but they did not found a significant association of total PUFAs [13] or n-3 PUFAs intake [14]. An experimental study reported that n-3 PUFAs in comparison to n-6 PUFAs shows inhibitory effects on BC [15].

Lipoproteins are the distributors of both endogenous and exogenous lipids across the tissues. It is therefore possible that lipoproteins can play a fundamental role in the progression of cancer via lipids supply to malignant cells and tumors. The level of plasma lipids reflects the dietary lipid intake in individuals. There are several reports of increased plasma lipid levels such as total lipids (TL), phospholipids (PL), triglycerides (TG), total cholesterol (TC) and low density lipoproteins (LDL-C) in BC patients [16]. Therefore, the present study was aimed to determine the possible effect of RT on serum fatty acids and lipid profiling in BC patients (before and after treatment), in comparison to healthy individuals.

## Methods

Blood serum samples were collected from Nuclear Institute of Medicine and Radiotherapy (NIMRA), Jamshoro, Pakistan. One hundred and thirty female patients with BC aged 25 to 65 years were enrolled before starting the radiotherapy during January, 2014 to July, 2015. The dietary habits of patients were almost same according to nutritionist because they were admitted at Nuclear Institute of Medicine Radiotherapy, Jamshoro, Pakistan for completing the therapy of 6 cycles. The female patients selected were those who carry BC, since 1 year and the tumor size of patients were also almost same. Firstly, we collected the blood sample before starting the radiotherapy, when a female patient was agreed for this procedure of treatment. After completion of all six cycles of radiotherapy, the blood samples of same female BC patients were collected as post treated patients. Those female BC patients who were treated with radiotherapy elsewhere and continued their therapy at NIMRA were excluded from the study. Those females who discontinued their radiotherapy due to any reason or having other complications due to radiotherapy and all male BC patients were excluded from the present study. We also collected fifty blood samples from untreated normal volunteer female subjects. The controls met the criteria of having negative personal and family history of any cancer disease, age and gender matched, non- smokers, having normal BMI (non- obese) were included in present study. The females with cardiovascular diseases, diabetes and hyperlipidemia were excluded from the study. All the participant females signed written informed consents before sample collection; they were also consented for publication. The study was carried out by following the regulations of Institutional Ethnical Committee, Institute of Biochemistry, University of Sindh, Jamshoro, Pakistan under their permission letter.

The blood samples from all participants were collected in fasting condition. Serum was separated and stored at −40 °C until analyzed further. Fatty acids and lipid profiling were performed by gas chromatograph 8700 (Perkin–Elmer Ltd., England) and Vital Scientific Microlab 300 (Germany), respectively. Fatty acid contents such as free fatty acids (FFAs) and total fatty acids (TFAs) were analyzed before and after RT. The FFAs and TFAs samples were prepared as per reported method [17]. Briefly, gas chromatograph, model 8700 fitted with non-bonded biscynopylsiloxane stationary-phase, polar capillary column Rt-2560(100 m × 0.25 mm) with a film thickness of 0.2 μm (Supelco, PA, USA) and flame ionization detector was used. Oxygen free nitrogen was used as a carrier gas at a flow rate of 3.5 ml/min. The initial temperature of the oven was adjusted at 120 °C/4 min, which was raised up to 220 °C for 20 min. The injector and detector temperature were set at 260 °C and 270 °C, respectively. 2 μl sample was injected from injector and peaks were measured by comparing with standards supplied by Fluka Chemika (Buchs, Switzerland). Thirteen fatty acids of three different groups are given as follows;

**SFAs:** C12:0 (lauric acid) C14:0 (myristic acid), C15:0 (pentadecyclic acid)C16:0 (palmitic acid), and C18:0 (Stearic acid).

**MUFAs:** C14:1 (myristoleic acid), C16:1 (palmitoleic acid), and C18:1 (oleic acid).

**PUFAs:** C18:2 (linoleic acid), C18:3 (α-linolenic acid), C20:3 (Dihomo-γ-linolenic acid), C20:4 (Arachidonic acid), and C22:6 (docosahexaenoic acid). The composition of FA’s was reported by relative percentage of the total peak area.

D5 Desaturase was calculated as 20:4n-6/20:3n-6 ratio. D6 Desaturase was calculated as 20:3n-6/18:2n-6 ratio [18]. Lipid profile contains total cholesterol (TC), triglycerides (TG), high density lipoprotein cholesterol (HDL-C), low density lipoprotein cholesterol (LDL-C) and very low density lipoprotein cholesterol (VLDL-C) which were analyzed by using kit method (Merck, Germany) in microlab 300 [19].

### Statistical analysis

The values obtained were expressed as mean ± SD. To compare data among the age groups ANOVA was applied and to compare the patients data with controls and patients data for comparison between pre and post treated, student’s t-test was applied by using SPSS 15.2 software. *p* value less than 0.05 was considered as significant.

## Results

**Fig. 1:**
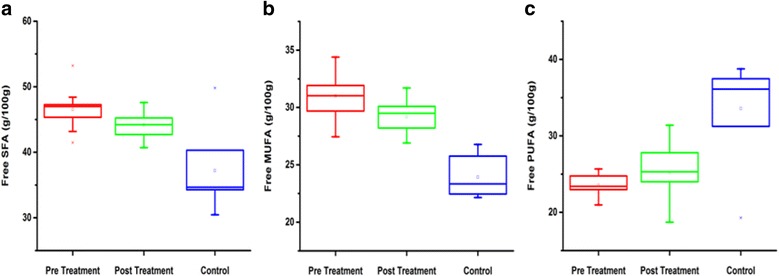
Detailed evaluation of free form of fatty acids. **a** Shows free SFAs level in pre-treated and post-treated as compared with control. **b** Shows free MUFAs level in pre and post-treatment patients as compared with control. **c** Shows Free PUFAs level between pre-post-treatment and control subjects

Free fatty acid levels with n-3 and n-6 classes of serum lipids in breast cancer patients (pre and post treated) were compared with controls (Table 1)(data is presented in Additional files 1, 4 and 9). The SFAs in serum lipids of BC patients (pre and post treated) were significantly increased, stearic acid (C18:0) and palmitic acid (C16:0) were the major SFAs (Fig. 1a). In MUFAs, a significant increase in the concentration of oleic acid (C18:1) was observed in BC patients (Fig. 1b). In PUFAs, decreased concentrations of linoleic acid (C18:2) and Arachidonic acid (C20:4) were observed in BC patients as compared to controls (Fig. 1c). Lower ratio of C18:2/C18:1, whereas, a decrease in n-3/n-6 ratio was observed in BC patients but improvement in n-3/n-6 was found after RT treatment. In addition, plasma activity of D6 desaturase was increased in the breast cancer patients while the activity of D5 desaturase was decreased (Table 1).

**Table 1:** Free fatty acid profile of BC patients (pre and post) in comparison with controls

Free fatty acids	Controls	Pre-treated BC patients	Post-treated BC patients
C-14: 0	1.26 ± 0.96	1.30 ± 0.30	0.81 ± 0.44
C-15: 0	ND	0.57 ± 0.24	ND
C-16: 0	18.17 ± 5.06	23.0 ± 2.86^*^	28.2 ± 2.47^*^
C-18: 0	13.89 ± 3.56	21.8 ± 2.00^*^	15.9 ± 1.76^¶^
C-14: 1	0.75 ± 0.49	1.86 ± 0.51	0.04 ± 0.01
C-16: 1	2.619 ± 1.37	1.85 ± 0.48	2.66 ± 0.96
C-18: 1	19.71 ± 2.276	27.3 ± 1.63^*^	26.5 ± 2.01^*^
C-18: 2 (n-6)	29.14 ± 3.28	20.2 ± 1.65^*^	19.84 ± 2.37*
C-18: 3 (n-3)	1.54 ± 0.79	1.21 ± 0.836^*^	0.76 ± 0.40*
C-20: 3 (n-6)	0.65 ± 0.3	0.39 ± 0.19	0.6 ± 0.47
C-20: 4 (n-6)	7.76 ± 2.40	1.09 ± 0.52^*^	2.7 ± 1.55^*¶^
C-22: 6 (n-3)	1.18 ± 0.79	0.47 ± 0.24	1.88 ± 1.74
C-18:0: C-18:1	0.719 ± 0.24	0.79 ± 0.06	0.605 ± 0.090
C-18:2: C-18:1	1.5 ± 0.28	0.074 ± 0.03	0.75 ± 0.118
C-18:3: C-18:1	0.071 ± 0.033	2.8 ± 0.97^*^	0.008 ± 0.002^*¶^
n-3: n-6	7.4 ± 1.41	2.80 ± 1.01^*^	3.43 ± 1.71*^¶^
Sat: unsat FA’s	35.2 ± 6.05	25.0 ± 1.34^*^	26.6 ± 3.47^*^
D5 Desaturase	21.2 ± 19.1	3.83 ± 3.4^*^	7.84 ± 1.2
D6 Desaturase	0.019 ± 0.009	0.03 ± 0.02	0.02 ± 0.013

Values represent mean ± standard deviation of triplicates. The different symbol on the same row indicates the Significant difference at *p* < 0.05. *p < 0.05 shows the comparison between pre and post treated BC patients with controls, ¶ *p* < 0.05 shows the comparison between pre-treated BC patients and post treated BC patients. Lauric acid (C12:0), Myristic acid (C14:1), myristoleic acid (C14:1), Pentadecyclic acid (C15:0), palmitic acid (C16:0), palmitoleic acid (C16:1), stearic acid (C18:0), oleic acid (C18:1), linoleic acid (C18:2), α-linolenic acid (C18:3), C20:3 (Dihomo-γ-linolenic acid), arachidonic acid (C20:4) and docosahexaenoic acid (DHA (C22:6). D5 Desaturase was calculated as 20:4n-6/20:3n-6 ratio. D6 Desaturase was calculated as 20:3n-6/18:2n-6 ratio. Sat: unsat FA’s shows saturated and unsaturated fatty acids ratio

**Fig. 2:**
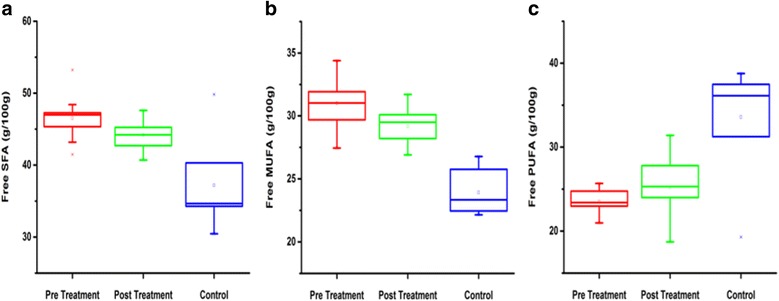
Detailed evaluation of total form of fatty acids. The SFAs (**a**), MUFAs (**b**) were elevated and PUFAs (**c**) was lower in Breast cancer (pre and post treated) patients in contrast with controls. The Significant variation (*P* < 0.05) was found in total fatty acids including SFAs and PUFAs

In addition, same results were observed in total fatty acids as well (Table 2)(data is presented﻿ in Additional files 2, 3 and 10). Total SFAs levels were significantly increased in BC (pre and post treated) patients as compared to controls (Fig. 2a). MUFAs were also elevated in BC patients as compared to controls (Fig. 2b). In contrast, PUFAs were reduced in BC (pre and post treated) patients as compared to controls (Fig. 2c). The Significant variation (*P* < 0.05) was observed in free fatty acids including SFAs and PUFAs.

**Table 2:** Total fatty acid profile of BC patients (pre and post) in comparison with controls

Total fatty acid	Controls	Pre-treated BC patients	Post-treated BC patients
C-12: 0	ND	0 ± 0	0.42 ± 0.04
C-14: 0	1.26 ± 0.96	1.34 ± 0.30	2.03 ± 1.06
C-15: 0	0.80 ± 0.13	0.47 ± 0.22	1.86 ± 1.18
C-16: 0	18.2 ± 5.06	22.3 ± 2.57^*^	20.7 ± 2.73^*^
C-18: 0	14.6 ± 3.56	23.1 ± 2.43^*^	22.9 ± 2.36^*^
C-14: 1	0.80 ± 0.74	1.19 ± 0.60	0.86 ± 0.42
C-16: 1	2.62 ± 1.37	1.72 ± 0.43^*^	1.34 ± 0.76^*^
C-18: 1	19.7 ± 2.276	25.4 ± 1.6^*^	24.6 ± 2.32^*^
C-18: 2 (n-6)	29.1 ± 3.28	20.64 ± 1.94^*^	22.1 ± 2.39^*^
C-18: 3 (n-3)	0.98 ± 0.76	1.69 ± 0.60^*^	2.36 ± 1.85^*^
C-20: 3 (n-6)	1.58 ± 0.87	0.55 ± 0.30	0.72 ± 0.69
C-20: 4 (n-6)	7.72 ± 2.42	1.24 ± 0.46^*^	1.59 ± 0.96^*^
C-22: 6 (n-3)	0.46 ± 0.30	0.51 ± 0.19	1.15 ± 0.89
C-18:0: C-18:1	0.72 ± 0.24	0.91 ± 0.10	0.93 ± 0.14
C-18:2: C-18:1	1.5 ± 0.28	0.82 ± 0.09	0.91 ± 0.14
C-18:3: C-18:1	0.05 ± 0.03	0.07 ± 0.02	0.10 ± 0.09
n-3: n-6	9.8 ± 3.99	3.4 ± 0.86^*^	4.62 ± 1.95^*^
Sat: unsat FA’s	41.0 ± 4.50	26.2 ± 2.02^*^	29.1 ± 3.22^*^
D5 Desaturase	7.75 ± 6.02	2.66 ± 1.25^*^	3.53 ± 3.45^*^
D6 Desaturase	0.026 ± 0.014	0.05 ± 0.03	0.03 ± 0.02

* *p* < 0.05 shows the comparison pre and post treated BC patients with controls, ¶ *p* < 0.05 shows the comparison of pretreated BC patients with post treated BC patients. Values represent mean ± standard deviation of triplicates. The different symbol on the same row indicates the Significant difference at *p* < 0.05. Lauric acid (C12:0), Myristic acid (C14:1), myristoleic acid (C14:1), Pentadecyclic acid (C15:0), palmitic acid (C16:0), palmitoleic acid (C16:1), stearic acid (C18:0), oleic acid (C18:1), linoleic acid (C18:2), α-linolenic acid (C18:3), C20:3 (Dihomo-γ-linolenic acid), arachidonic acid (C20:4), and docosahexaenoic acid (DHA (C22:6).D5 Desaturase was calculated as 20:4n-6/20:3n-6 ratio.D6 Desaturase was calculated as 20:3n-6/18:2n-6 ratio. Sat: unsat FA’s shows saturated and unsaturated fatty acids ratio

**Fig. 3:**
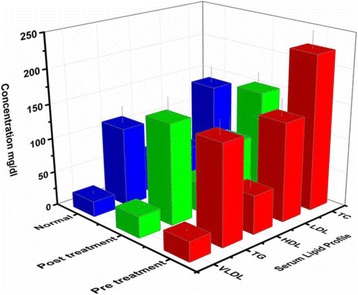
Serum lipid profile of pre and post treatment BC patients in comparison with control: The figure pretreated that the patients have significant positive association of high level of TC(224.4 ± 25.21), low level of HDL-C(55.96 ± 16.97) and high level of LDL-C (142.9 ± 25.04) with Breast cancer disease (Red bar). After treatment the level of TC and LDL-C decreases significantly. (Green Bar)

**Fig. 4:**
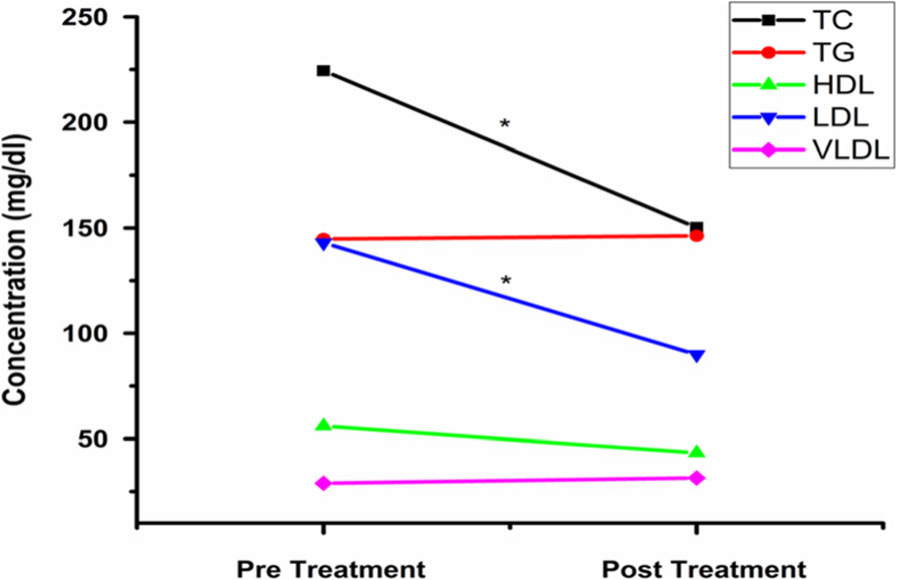
Showed the comparison between pre-treated and post-treated BC patients. Represented that TC and LDL levels are decreased in post-treatment as compared to post-treatment BC patients. While as, TG, HDL and VLDL levels showed no significant change

Levels of TC, LDL-C, HDL-C, VLDL and TG were also examined (Table 3). Where, serum TC and LDL-C in pretreated BC patients were significantly increased as compared to controls (Fig. 3), while, after RT treatment TC and LDL-C levels were decreased significantly (Fig. 4)(data is ﻿presented in Additional files 5, 6, 7 and 8). Whereas TG and VLDL-C increased in post treated BC patients as compared with controls but within normal ranges. The non-significant level of HDL-C and high level of TC were also found affected on TC: HDL-C ratio (Table 3).

**Table 3:** Comparison of lipid profile in BC (pre and post treated) patients with controls

Lipid parameter (mg/dL)	Controls *n* = 50	Pre-treated BC patients *n* = 130	Post-treated BC patients *n* = 130
TC (<200)	137.88 ± 24.45	224.4 ± 25.21^*^	150.2 ± 22.19^* ¶^
TG (<150)	114.42 ± 24.74	144.7 ± 21.28	146.2 ± 27.42
HDL (<40)	56.56 ± 14.77	55.96 ± 16.97	43.36 ± 5.62 ^¶^
LDL(<100)	54.16 ± 12.71	142.9 ± 25.04^*^	89.83 ± 26.68^* ¶^
TL (450-1000)	509.72 ± 37.97	718.1 ± 53.74^*^	579.7 ± 55.59^* ¶^
VLDL(<30)	22.67 ± 4.75	28.95 ± 4.25^*^	31.49 ± 5.71^*^
TC:HDL(4.0)	2.712 ± 0.84	4.48 ± 1.60	3.54 ± 0.72
TC:LDL (1-6)	2.70 ± 0.91	1.59 ± 0.17^*^	1.78 ± 0.53 ^*¶^
HDL:LDL (0.5)	1.06 ± 0.45	0.39 ± 0.145^*^	0.51 ± 0.14 ^*^

*p < 0.01 shows the comparison between pre-treated and post treated BC patients with controls, ¶ *p* < 0.01 shows the comparison between pre-treated BC patients with post treated BC patients

## Discussion

In present study, the effective role of RT was examined. Significant differences were observed in FAs and lipid profile in serum samples from patients with BC before and after RT treatment. The levels of PUFAs were increased, whereas, TC and LDL-C levels were significantly decreased after the RT treatment. SFAs levels were also found to be associated with the risk of BC (Fig. 1a–c and 2a–c). In SFAs, stearic and palmitic acid while, in MUFAs oleic acid were found significantly increased in BC (pre and post treated). D6 desaturase activity was higher in BC patients, while the n-3/n-6 ratio and D5 desaturase activity were lower in BC patients compared to controls (Tables 1 and 2).

Metabolic pathways as well as the dietary intake influence the FAs composition. Arachidonic acid originates from both diet and elongation desaturation process of its precursor, linoleic acid. The D5 and D6 desaturases are the key enzymes not only for this pathway, but also play a role in the n-3 fatty acid pathway. So, the indirect information was collected from the lipid composition analysis which provides a suitable and simple model for studying FAs metabolism [20]. Lipid autacoids which produced endogenously, locally acting eicosanoids play a key role in the tissue homeostasis and have recently implicated in cancer. These eicosanoids were generated by distinct enzymatic systems initiated by cyclooxygenase, cytochrome p450 and lipooxygenase [21, 22]. In another study, excess of SFAs were found cytotoxic to BC cells [23]. Hilvo et al. (2011) measured the lipid composition in tissue membrane of BC and observed the high concentration of phosphatidylcholine, which is a palmitate containing species in BC in comparison to normal breast tissues [24]. Results of the previous study showed increased concentrations of lumina A and B subtypes of all phospholipid (PL) classes like ethanolamine, phosphatidyl inositol and choline in BC. In these PL, acyl group analysis showed a high level of C16:0 and C18:1 FAs due to increasing in de novo synthesis as their source. High concentration of SFAs content in cultured cell membranes decreased the permeability of cell membrane, sensitivity and number of insulin receptors, due to which insulin resistance increased. Insulin resistance is related to the increase in free IGF-I and decrease in IGF binding protein I. According to epidemiological studies, increased free IGF-I and decreased IGF protein I increase the risk of BC. This phenomenon has combined effect with a high level of estradiol stored in fatty acid esters, which may enhance BC [25]. SFAs can also increase the risk of BC by increasing insulin resistance [26].

In another study, the high consumption of animal fat, meat and SFAs was found to be associated with increased risk of BC [27, 28]. A meta-analysis of 10 case control studies [13] and some animal studies, [29] have shown that MUFAs play a positive role in the pathogenesis of BC. MUFAs also have different effect on BC [30]. In addition, our data advocates that there is no correlation between PUFAs levels and BC. Whereas, Serini et al. [31] proposed that n-3 PUFA plays a significant role in the incidence of BC by the growth of tumor cells and increase cell replication process, by interfering cell cycle. Some studies show that n-3 PUFAs prevent BC by influencing the enzyme activity [32, 33]. Some of the case-control studies have reported positive [33, 34] relation of n-6 fatty acids intake with breast cancer risk. n-6 fatty acids by competition with n-3 fatty acids to produce eicosanoids increase the risk of breast cancer and also because of having many double bonds are easily oxidized and enhance cellular damage [35]. Moreover, a meta-analysis of cohort studies [36] and also a systematic review [37] showed an inverse association of n-3 fatty acids with breast cancer risk. In this mentioned meta-analysis very long chain n-3 PUFAs intake, which was estimated by using the composition of fatty acids in biological samples such as adipose tissue, erythrocyte membranes, serum and plasma showed a protective effect on breast cancer [36]. The low ratio of n-3/n-6 PUFAs promotes the pathogenesis of BC [38].

In the present study, high level of TC and LDL-C were observed before RT, but, after treatment TC and LDL-C levels were decreased, significantly (Fig. 4). According to a study, significant benefits of radiotherapy were reported previously. Overall, 16% absolute decrease in the recurrence of BC and 4% decrease in the death rate by BC were observed [2]. Present study showed a strong association of TC and LDL-C to BC (Table 3). Lipids are known to play an important role in tumor development and progression, as Cancer cells need lipids for membrane biogenesis and protein modifications [39]. The cancer cells that are not rapidly proliferating require increased amounts of lipids for enhanced signaling and resistance to apoptosis [6]. Similar results were found by other researchers and proposed that high serum TC level plays a significant role in Breast carcinogenesis [40–43]. Elkhadrawy et al. [43] found a contrary association of TC in BC patients, but LDL-C level was also found increased in BC patients in this study. High dietary fat intakes have been found to be positively related to breast cancer in many epidemiological studies. High concentration of plasma LDL-C is more vulnerable to oxidation, cause of higher lipid peroxidation in BC patients [44].

In our study, no association between HDL-C and BC was observed (Fig. 3). Similarly, Bhat et al. [45] also reported no significant changes for HDL-C in BC patients as compared to controls. Inverse association for HDL-C was found by Borelli R, et al. [46] and argued that HDL-C is a biochemical index which can be associated with increased risk of BC. BC patients whom were reported with a high level of blood LDL-C were also found with higher levels of oxidized LDL-C. The oxidized LDL-C is related with increased risk of BC, while HDL-C is less susceptible to peroxidation due to its lipids and apoprotein content. Hence, HDL do not produce reactive oxygen species and acts as an anti-carcinogen [47].TG was not significantly increased in BC patients in our findings, while another study found a significant increase in TG levels in BC patients [48].

## Conclusion

In conclusion, RT is an effective treatment and plays an important role in the lipid profile and fatty acid management in most patients with BC. The improvement in n-3/n-6 was found after treatment, as evident from the results of post treated patients. After treatment, the n-3/n-6 ratio was increased by 35%. RT benefits depend on the advances in both surgery and systemic treatment. It also contributes benefit for the treatment of BC to reduce breast cancer mortality rates. So, it is expected that RT will play significant role in the health care system.

## Additional files

Additional file 1: Serum free fatty acids in pre treated BC patients. (PDF 165 kb)

Additional file 2: Serum total fatty acid concentrations in Pre treated BC patients. (PDF 166 kb)

Additional file 3: Serum total fatty acid concentrations in controls. (PDF 158 kb)

Additional file 4: Serum free fatty acid concentrations in controls. (PDF 158 kb)

Additional file 5: Serum lipid profile of pre treated BC patients. (PDF 162 kb)

Additional file 6: Serum lipid profile of post treated BC patients. (PDF 154 kb)

Additional file 7: Serum lipid profile of controls. (PDF 232 kb)

Additional file 8: GC-FID Chromatogram showing serum total fatty acid profile of pre-treated BC patients. (PDF 233 kb)

Additional file 9: Serum free fatty acids in post-treated BC patients. (PDF 159 kb)

Additional file 10: Serum total fatty acids in post treated BC patients. (PDF 402 kb)

## Abbreviations

FAMEs: Fatty acid methyl esters
FAs: Fatty acids
FID: Flame ionization detector
GC: Gas chromatography
HDL-C: High density lipoprotein cholesterol
LDL-C: Low density lipoprotein cholesterol
MUFAs: Monounsaturated fatty acids
PUFAs: Poly unsaturated fatty acids
SFAs: Saturated fatty acids
TC: Total cholesterol
TG: Triglycerides
VLDL-C: Very low density lipoprotein cholesterol
