# Supplementary material for: Radiotherapy improves serum fatty acids and lipid profile in breast cancer
Source: Lipids Health Dis. 2017 May 18;16:92. doi: 10.1186/s12944-017-0481-y (PMC5437547; doi:10.1186/s12944-017-0481-y)
Supplement: Supplementary file 1 — Serum free fatty acids in pre treated BC patients. (PDF 165 kb) [file 12944_2017_481_MOESM1_ESM.pdf]

Post Free Fatty acid

Name of pa	C-12:0	C - 14: 0	C-15:0	C - 16 : 0	C - 18 : 0	Total SFA	C 14:1	C - 16 : 1
Post 1	0	0	0	26.34	18.9	45.24	0	2.3
Post 2	0	0	0	25.1	17.5	42.6	0	1.9
Post 3	0	0	0	28.7	15.5	44.2	0	2.1
Post 4	0	0	0	31.9	13.7	46	0	3.4
Post 5	0	0	0	31.8	15.8	47.6	0	4.2
Post 6	0	0	0	26.5	14.2	40.7	0	3.3
Post 7	0	0	0	28.9	13.3	42.2	0	3.1
Post 8	0	0	0	25.5	17.2	42.7	0	2.6
Post 9	0	0	0	30.1	14.7	44.8	0	1.9
Post 10	0	0	0	29.34	15	44.34	0	2.2
Post 11	0	0.33	0	27.98	16.1	44.08	0.03	1.76
post 12	0	1.2	0	24.54	18.34	42.88	0	4.5
post 13	0	0.91	0	30.35	16.87	47.22	0	1.4
post 14	0	0	0	26.34	18.9	45.24	0	2.3
post 15	0	0	0	25.1	17.5	42.6	0	1.9
post 16	0	0	0	28.7	15.5	44.2	0	2.1
post 17	0	0	0	31.9	13.7	46	0	3.4
post 18	0	0	0	31.8	15.8	47.6	0	4.2
post 19	0	0	0	26.5	14.2	40.7	0	2.2
post 20	0	0	0	28.9	13.3	42.2	0.05	1.75
post 21	0	0	0	25.5	17.2	42.7	0	4.5
post 22	0	0	0	30.1	14.7	44.8	0	1.4
post 23	0	0	0	29.34	15	44.34	0	2.1
post 24	0	0	0	27.98	16.1	44.08	0	3.4
post 25	0	0	0	24.54	18.34	42.88	0	4.2
post 26	0	0	0	30.35	16.87	47.22	0	3.3
post 27	0	0	0	25.5	17.2	42.7	0	3.1
post 28	0	0	0	30.1	14.7	44.8	0	2.6
post 29	0	0	0	29.34	15	44.34	0	1.9
post 30	0	0	0	31.9	13.7	46	0.04	1.77
Mean	0	0.813333	0	28.23462	15.93154	44.19692	0.04	2.666154
Stdev	0	0.442982	0	2.476559	1.757877	2.004127	0.01	0.962579

C - 18 : 1	MUFA	C - 18 : 2	C - 18 : 3	c-20:2	C - 20 : 4	C - 22:6	PUFA	C18:0/C18:1
26.2	28.5	20	0.9	0	4.6	5.9	31.4	0.72137405
27.6	29.5	21.3	0	0	4.8	2.3	28.4	0.63405797
29.6	31.7	22.5	0	0	3.5	1.8	27.8	0.52364865
24.8	28.2	19.9	0	2.6	2.6	2.8	27.9	0.55241935
26.9	31.1	18.6	0	0	3.8	2.9	25.3	0.58736059
24.2	27.5	23.5	0	0	1.6	0.9	26	0.58677686
27	30.1	19.3	0	0	2.6	3.6	25.5	0.49259259
24.3	26.9	18.5	0	1.01	4.9	0	24.41	0.70781893
28.3	30.2	17.4	0	3.9	2.4	0	23.7	0.51943463
26.3	28.5	15.3	0.2	2.8	1.7	0.6	20.6	0.57034221
28.3	30.1	17.6	0	0	0.67	0.45	18.72	0.56890459
22.7	27.2	22	0	0	2	0	24	0.80792952
28.4	29.8	22.1	0	0	0	2	24.1	0.59401408
26.2	28.5	20	0.9	0	4.6	5.9	31.4	0.72137405
27.6	29.5	21.3	0	0	4.8	2.3	28.4	0.63405797
29.6	31.7	22.5	0	0	3.5	1.8	27.8	0.52364865
24.8	28.2	19.9	0	2.6	2.6	2.8	27.9	0.55241935
26.9	31.1	18.6	0	0	3.8	2.9	25.3	0.58736059
26.3	28.5	15.3	0.2	2.8	1.7	0.6	20.6	0.53992395
28.3	30.1	17.6	0	0	0.67	0.45	18.72	0.46996466
22.7	27.2	22	0	0	2	0	24	0.75770925
28.4	29.8	22.1	0	0	0	2	24.1	0.51760563
29.6	31.7	23.5	0	0	1.6	0.9	26	0.50675676
24.8	28.2	19.3	0	0	2.6	3.6	25.5	0.64919355
26.9	31.1	18.5	0	1.01	4.9	0	24.41	0.68178439
24.2	27.5	17.4	0	3.9	2.4	0	23.7	0.69710744
27	30.1	17.6	0	0	0.67	0.45	18.72	0.63703704
24.3	26.9	22	0	0	2	0	24	0.60493827
28.3	30.2	22.1	0	0	0	2	24.1	0.53003534
28.3	30.1	20	0.9	0	4.6	5.9	31.4	0.48409894
26.50769	29.17692	19.84615	0.666667	2.503333	2.705385	1.788462	25.21769	0.60512877
2.018853	1.502306	2.37368	0.404145	1.447423	1.5527	1.740229	3.318389	0.09071327

n3/n6	c18:2/C18:	C18:3/C18:1	sat/unsat
5.795	0.763359	0.034351145	32.98737
4.907981	0.771739	0	29.84407
3.58	0.760135	0	29.19432
5.340704	0.802419	0	29.53121
3.955914	0.69145	0	26.83055
1.638298	0.971074	0	27.48
2.786528	0.714815	0	26.90199
5.91	0.761317	0	25.99736
6.3	0.614841	0	25.18344
4.739216	0.581749	0.007604563	22.15579
0.695568	0.621908	0	20.18445
2	0.969163	0	25.57647
0.090498	0.778169	0	25.68456
5.795	0.763359	0.034351145	32.98737
4.907981	0.771739	0	29.84407
3.58	0.760135	0	29.19432
5.340704	0.802419	0	29.53121
3.955914	0.69145	0	26.83055
4.739216	0.581749	0.007604563	22.02807
0.695568	0.621908	0	20.12199
2	0.969163	0	25.56985
0.090498	0.778169	0	25.60336
1.638298	0.793919	0	27.39874
2.786528	0.778226	0	27.06312
5.91	0.687732	0	25.78878
6.3	0.719008	0	25.41709
0.695568	0.651852	0	20.1386
2	0.90535	0	25.66543
0.090498	0.780919	0	25.56821
5.795	0.706714	0.03180212	32.92824
3.672285	0.754011	0.008123	26.64102
2.068902	0.118593	0.002	3.478717
