# Supplementary material for: Radiotherapy improves serum fatty acids and lipid profile in breast cancer
Source: Lipids Health Dis. 2017 May 18;16:92. doi: 10.1186/s12944-017-0481-y (PMC5437547; doi:10.1186/s12944-017-0481-y)
Supplement: Supplementary file 2 — Serum total fatty acid concentrations in Pre treated BC patients. (PDF 166 kb) [file 12944_2017_481_MOESM2_ESM.pdf]

Post total

Name of pa	C-12:0	C - 14: 0	C-15:0	C - 16 : 0	C - 18 : 0	Total SFA	C 14:1	C - 16 : 1
post 1	0	3.36	3.36	18.2	22.7	47.62	0	0
post 2	0	2.65	2.01	23	23	50.66	0	0
post 3	0	0	0	22.34	21.54	43.88	0	1.12
post 4	0.4	3.592	3.59	14.27	25.35	47.202	1.04	1.25
post 5	0	0	0	18.31	25.7	44.01	0.55	2.4
post 6	0	0	0	24.3	22.4	46.7	0.87	1.15
post 7	0	1.15	1.5	19.4	25.5	47.55	0	2.4
post 8	0.42	0	0	22.03	17.21	39.66	0	1.05
post 9	0.4	0	0	24.32	22.01	46.73	0.86	1.11
post 10	0.5	1.25	1.25	22.68	23.67	49.35	0.55	1.01
post 11	0.4	1.42	1.1	19.73	26.12	48.77	0.3	2
p0st 12	0	1.5	0.2	20.12	22.21	44.03	1.9	2.3
post 13	0	1	0.8	20.8	20.6	43.2	0.8	1.6
post 14	0	0	0	24.3	22.4	46.7	0.87	1.15
post 15	0	1.15	1.5	19.4	25.5	47.55	0	2.4
post 16	0.42	0	0	22.03	17.21	39.66	0	1.05
post 17	0.4	0	0	24.32	22.01	46.73	0.86	1.11
post 18	0	3.36	3.36	18.2	22.7	47.62	0	0
post 19	0	2.65	2.01	23	23	50.66	0	0
post 20	0	0	0	22.34	21.54	43.88	0	1.12
post 21	0.4	3.592	3.59	14.27	25.35	47.202	1.04	1.25
post 22	0	0	0	18.31	25.7	44.01	0.55	2.4
post 23	0.5	1.25	1.25	22.68	23.67	49.35	0.55	1.01
post 24	0.4	1.42	1.1	19.73	26.12	48.77	0.3	2
post 25	0	1.5	0.2	20.12	22.21	44.03	1.9	2.3
post 26	0	1	0.8	20.8	20.6	43.2	0.8	1.6
post 27	0.4	3.592	3.59	14.27	25.35	47.202	1.04	1.25
post 28	0	0	0	18.31	25.7	44.01	0.55	2.4
post 29	0	0	0	24.3	22.4	46.7	0.87	1.15
post 30	0	1.15	1.5	19.4	25.5	47.55	0	2.4
Mean	0.424	2.0342	1.867	20.73077	22.92385	46.10477	0.86	1.337692
Stdev	0.040879	1.060894	1.183357	2.828505	2.460357	3.004634	0.424735	0.795908

Fatty acid

C - 18 : 1	MUFA	C - 18 : 2	C - 18 : 3	c-20:2	C - 20 : 4	C - 22:6	PUFA	C18:0/C18:
20.54	20.54	24.42	7.42	0	0	0	31.84	1.105161
20.78	20.78	19.5	3.43	2.2	3.43	0	28.56	1.106833
25.32	26.44	23.01	2.15	0	1.8	2.7	29.66	0.850711
26.78	29.07	20.08	3.05	0.34	0.11	0.12	23.7	0.946602
27	29.95	23.21	3.14	1.9	1.7	0	29.95	0.951852
25.98	28	20.12	2.16	0	1.34	0.38	24	0.862202
24.62	27.02	21.41	0	0	1.9	1.12	24.43	1.035743
27.05	28.1	27.04	0	0	3	2.2	32.24	0.636229
24.86	26.83	24.05	1.03	0.65	0.71	0	26.44	0.885358
26.32	27.88	18.73	1.27	1.2	1.57	0	22.77	0.899316
24.3	26.6	19.4	3.2	1.9	1.9	0.2	26.6	1.074897
20.77	24.97	24.3	2.8	1.5	0.9	1.5	31	1.069331
26	28.4	22.3	1.1	0.9	2.3	1.8	28.4	0.792308
25.98	28	20.12	2.16	0	1.34	0.38	24	0.862202
24.62	27.02	21.41	0	0	1.9	1.12	24.43	1.035743
27.05	28.1	27.04	0	0	3	2.2	32.24	0.636229
24.86	26.83	24.05	1.03	0.65	0.71	0	26.44	0.885358
20.54	20.54	24.42	7.42	0	0	0	31.84	1.105161
20.78	20.78	19.5	3.43	2.2	3.43	0	28.56	1.106833
25.32	26.44	23.01	2.15	0	1.8	2.7	29.66	0.850711
26.78	29.07	20.08	3.05	0.34	0.11	0.12	23.7	0.946602
27	29.95	23.21	3.14	1.9	1.7	0	29.95	0.951852
26.32	27.88	18.73	1.27	1.2	1.57	0	22.77	0.899316
24.3	26.6	19.4	3.2	1.9	1.9	0.2	26.6	1.074897
20.77	24.97	24.3	2.8	1.5	0.9	1.5	31	1.069331
26	28.4	22.3	1.1	0.9	2.3	1.8	28.4	0.792308
26.78	29.07	20.08	3.05	0.34	0.11	0.12	23.7	0.946602
27	29.95	23.21	3.14	1.9	1.7	0	29.95	0.951852
25.98	28	20.12	2.16	0	1.34	0.38	24	0.862202
24.62	27.02	21.41	0	0	1.9	1.12	24.43	1.035743
24.64	26.50615	22.12077	2.365385	1.344	1.589231	1.152	27.66077	0.939734
2.410709	2.883937	2.490095	1.925697	0.690993	0.960066	0.894487	3.243459	0.139087

n3/n6	c18:2/C18:	C18:3/C18:1	sat/unsat
7.42	1.1889	0.36124635	34.1584
9.06	0.938402	0.16506256	30.99792
4.06734	0.908768	0.08491311	31.31961
3.505976	0.749813	0.11389096	25.32374
6.74	0.85963	0.1162963	31.41945
3.518887	0.774442	0.08314088	25.66786
1.952312	0.869618	0	26.18981
3.081361	0.99963	0	33.65139
2.39	0.967418	0.04143202	28.18171
4.04	0.711626	0.04825228	24.54009
7.010309	0.798354	0.13168724	28.43346
5.261728	1.169957	0.13480982	32.76332
4.380717	0.857692	0.04230769	29.92113
3.518887	0.774442	0.08314088	25.66786
1.952312	0.869618	0	26.18981
3.081361	0.99963	0	33.65139
2.39	0.967418	0.04143202	28.18171
7.42	1.1889	0.36124635	34.1584
9.06	0.938402	0.16506256	30.99792
4.06734	0.908768	0.08491311	31.31961
3.505976	0.749813	0.11389096	25.32374
6.74	0.85963	0.1162963	31.41945
4.04	0.711626	0.04825228	24.54009
7.010309	0.798354	0.13168724	28.43346
5.261728	1.169957	0.13480982	32.76332
4.380717	0.857692	0.04230769	29.92113
3.505976	0.749813	0.11389096	25.32374
6.74	0.85963	0.1162963	31.41945
3.518887	0.774442	0.08314088	25.66786
1.952312	0.869618	0	26.18981
4.802202	0.90725	0.10177225	29.12455
2.147698	0.147241	0.09357019	3.225224
