# Supplementary material for: Radiotherapy improves serum fatty acids and lipid profile in breast cancer
Source: Lipids Health Dis. 2017 May 18;16:92. doi: 10.1186/s12944-017-0481-y (PMC5437547; doi:10.1186/s12944-017-0481-y)
Supplement: Supplementary file 3 — Serum total fatty acid concentrations in controls. (PDF 158 kb) [file 12944_2017_481_MOESM3_ESM.pdf]

Pre Free Fatty acid

Pre 1	C-14:0	C-15:0	C - 16 : 0	C - 18 : 0	Total SFA	C 14:1	C - 16 : 1	C - 18 : 1
Pre 2	1.5	0	23.58	21.95	47.03	2.01	1.36	26.32
Pre 3	1.3	0	21.33	24.36	46.99	1.8	2.1	28.02
Pre 4	1.8	0.3	25.2	20.9	48.2	1.3	1.8	29.3
Pre 5	1.2	0.2	25.9	19.8	47.1	2.5	1.2	27.9
Pre 6	0.9	0.5	20.1	22.4	43.9	1.2	1.23	26.9
Pre 7	1.56	0	21.2	24.5	47.26	1.87	1.65	27.32
Pre 8	1.42	0.33	24.36	19.9	46.01	1.31	2.8	23.34
Pre 9	1.63	0	20.2	23.5	45.33	2.1	1.45	29.52
Pre 10	1.24	0.85	25.68	18.72	46.49	2.4	1.85	27.52
Pre 11	0.98	0.82	18.32	21.36	41.48	1.31	2	25.98
Pre 12	0.87	0.45	20.54	21.32	43.18	1.85	2.13	26.3
Pre 13	1.75	0.63	26.23	19.8	48.41	1.74	1.98	27.3
Pre 14	1.32	0.2	26.9	24.8	53.22	2.9	2.52	28.98
Pre 15	1.56	0.5	20.1	22.4	43.9	1.2	1.23	26.9
Pre 16	1.42	0	21.2	24.5	47.26	1.87	1.65	27.32
Pre 17	1.63	0.33	24.36	19.9	46.01	1.31	2.8	23.34
Pre 18	1.24	0	20.2	23.5	45.33	2.1	1.45	29.52
Pre 19	1.5	0.85	25.68	18.72	46.49	2.4	1.85	27.52
Pre 20	1.3	0.82	18.32	21.36	41.48	1.31	2	25.98
Pre 21	1.8	0.45	20.54	21.32	43.18	1.85	2.13	26.3
Pre 22	1.2	0.63	26.23	19.8	48.41	1.74	1.98	27.3
Pre 23	0.9	0.2	26.9	24.8	53.22	2.9	2.52	28.98
Pre 24	0.98	0	23.58	21.95	47.03	2.01	1.36	26.32
Pre 25	0.87	0	21.33	24.36	46.99	1.8	2.1	28.02
Pre 26	1.75	0.3	25.2	20.9	48.2	1.3	1.8	29.3
Pre 27	1.32	0.2	25.9	19.8	47.1	2.5	1.2	27.9
Pre 28	1.63	0.33	24.36	19.9	46.01	1.31	2.8	23.34
Pre 29	1.24	0	20.2	23.5	45.33	2.1	1.45	29.52
Pre 30	0.98	0.85	25.68	18.72	46.49	2.4	1.85	27.52
Mean	1.30324	0.487	23.04154	21.79308	46.50769	1.868462	1.851538	27.28462
Stdev	0.30123	0.244349	2.864608	2.009268	2.854036	0.519164	0.481419	1.63858

MUFA	C - 18 : 2	C - 18 : 3	c-20:2	C - 20 : 4	C - 22:6	PUFA	C18:0/C18:	n3/n6
29.69	19.5	1.9	0.74	1.01	0.54	23.69	0.833967	3.677692
31.92	19.9	2.5	0.63	1.3	0.42	24.75	0.869379	4.451106
32.4	21.2	1.9	0	1.6	0.65	25.35	0.713311	3.53066
31.6	23.9	0	0.68	0	0.22	24.8	0.709677	0.689205
29.33	18.5	1.4	0.24	1.2	0.21	21.55	0.832714	2.851351
30.84	20.1	0.8	0.32	1.05	0.71	22.98	0.896779	2.205323
27.45	21.3	0.7	0.41	0.99	0	23.4	0.852614	2.1
33.07	19.52	0.89	0.8	1.56	0.87	23.64	0.79607	3.29457
31.77	22.2	1.2	0	1.67	0.61	25.68	0.680233	2.897477
29.29	20.2	0	0.75	0.96	0.65	22.56	0.822171	1.742178
30.28	17.3	2.7	0.11	0.32	0.55	20.98	0.810646	3.161792
31.02	19.32	1.6	0	1.85	0.35	23.12	0.725275	3.468116
34.4	20.3	1.2	0.43	0.77	0.36	23.06	0.855763	2.417734
29.33	18.5	1.4	0.24	1.2	0.21	21.55	0.832714	2.851351
30.84	20.1	0.8	0.32	1.05	0.71	22.98	0.896779	2.205323
27.45	21.3	0.7	0.41	0.99	0	23.4	0.852614	2.1
33.07	19.52	0.89	0.8	1.56	0.87	23.64	0.79607	3.29457
31.77	22.2	1.2	0	1.67	0.61	25.68	0.680233	2.897477
29.29	20.2	0	0.75	0.96	0.65	22.56	0.822171	1.742178
30.28	17.3	2.7	0.11	0.32	0.55	20.98	0.810646	3.161792
31.02	19.32	1.6	0	1.85	0.35	23.12	0.725275	3.468116
34.4	20.3	1.2	0.43	0.77	0.36	23.06	0.855763	2.417734
29.69	19.5	1.9	0.74	1.01	0.54	23.69	0.833967	3.677692
31.92	19.9	2.5	0.63	1.3	0.42	24.75	0.869379	4.451106
32.4	21.2	1.9	0	1.6	0.65	25.35	0.713311	3.53066
31.6	23.9	0	0.68	0	0.22	24.8	0.709677	0.689205
27.45	21.3	0.7	0.41	0.99	0	23.4	0.852614	2.1
33.07	19.52	0.89	0.8	1.56	0.87	23.64	0.79607	3.29457
31.77	22.2	1.2	0	1.67	0.61	25.68	0.680233	2.897477
31.00462	20.24923	1.291538	0.512	1.098462	0.472308	23.50462	0.799892	2.806708
1.829843	1.658445	0.836519	0.232574	0.529825	0.240352	1.388576	0.069823	0.975875

c18:2/C18:	C18:3/C18:1	sat/Unsat
0.740881	0.07218845	25.27404
0.710207	0.089221984	26.22212
0.723549	0.064846416	26.83765
0.856631	0	26.29051
0.687732	0.05204461	23.04676
0.735725	0.029282577	24.51243
0.912596	0.029991431	25.07614
0.661247	0.030149051	25.01073
0.806686	0.043604651	27.14333
0.777521	0	23.97618
0.657795	0.102661597	22.40602
0.707692	0.058608059	24.68061
0.700483	0.041407867	24.60709
0.687732	0.05204461	23.04676
0.735725	0.029282577	24.51243
0.912596	0.029991431	25.07614
0.661247	0.030149051	25.01073
0.806686	0.043604651	27.14333
0.777521	0	23.97618
0.657795	0.102661597	22.40602
0.707692	0.058608059	24.68061
0.700483	0.041407867	24.60709
0.740881	0.07218845	25.27404
0.710207	0.089221984	26.22212
0.723549	0.064846416	26.83765
0.856631	0	26.29051
0.912596	0.029991431	25.07614
0.661247	0.030149051	25.01073
0.806686	0.043604651	27.14333
0.744519	0.047231284	25.08267
0.075529	0.030742635	1.349545
