# Supplementary material for: Radiotherapy improves serum fatty acids and lipid profile in breast cancer
Source: Lipids Health Dis. 2017 May 18;16:92. doi: 10.1186/s12944-017-0481-y (PMC5437547; doi:10.1186/s12944-017-0481-y)
Supplement: Supplementary file 4 — Serum free fatty acid concentrations in controls. (PDF 158 kb) [file 12944_2017_481_MOESM4_ESM.pdf]

Pre Total Fatty acid

Pre 1	C - 14: 0	C-15:0	C - 16 : 0	C - 18 : 0	Total SFA	C 14:1	C - 16 : 1	C - 18 : 1
Pre 2	1.5	0	23.58	21.95	47.03	0.62	1.36	26.32
Pre 3	1.3	0	21.33	24.36	46.99	1.1	2.1	24.6
Pre 4	1.8	0.3	25.2	20.9	48.2	1.2	1.8	25.3
Pre 5	1.2	0.2	19.8	25.9	47.1	0.5	1.2	23.7
Pre 6	0.9	0.5	20.1	22.4	43.9	1.2	1.36	22.3
Pre 7	1.56	0	21.2	24.5	47.26	0.87	1.65	24.3
Pre 8	1.42	0.33	24.36	19.9	46.01	1.31	0.98	23.34
Pre 9	1.63	0	24.32	27.08	53.03	2.1	1.45	27.52
Pre 10	1.24	0.85	19.68	24.72	46.49	0.68	1.85	26.31
Pre 11	0.98	0.82	18.32	21.36	41.48	0.31	1.96	25.98
Pre 12	0.87	0.45	20.54	21.32	43.18	1.85	2.13	26.3
Pre 13	1.75	0.63	26.23	19.8	48.41	1.74	1.98	27.3
Pre 14	1.32	0.2	24.9	25.8	52.22	2.1	2.52	26.98
Pre 15	0.9	0.5	20.1	24.5	47.26	0.87	1.65	24.3
Pre 16	1.56	0	21.2	19.9	46.01	1.31	0.98	23.34
Pre 17	1.42	0.33	24.36	27.08	53.03	2.1	1.45	27.52
Pre 18	1.63	0	24.32	24.72	46.49	0.68	1.85	26.31
Pre 19	1.24	0.85	19.68	21.95	47.03	0.62	1.36	26.32
Pre 20	1.5	0	23.58	24.36	46.99	1.1	2.1	24.6
Pre 21	1.3	0	21.33	20.9	48.2	1.2	1.8	25.3
Pre 22	1.8	0.3	25.2	25.9	47.1	0.5	1.2	23.7
Pre 23	1.2	0.2	19.8	22.4	43.9	1.2	1.36	22.3
Pre 24	0.98	0.82	18.32	21.36	41.48	0.31	1.96	25.98
Pre 25	0.87	0.45	20.54	21.32	43.18	1.85	2.13	26.3
Pre 26	1.75	0.63	26.23	19.8	48.41	1.74	1.98	27.3
Pre 27	1.32	0.2	24.9	25.8	52.22	2.1	2.52	26.98
Pre 28	1.2	0.2	19.8	22.4	43.9	1.2	1.36	22.3
Pre 29	0.9	0.5	20.1	24.5	47.26	0.87	1.65	24.3
Pre 30	1.56	0	21.2	19.9	46.01	1.31	0.98	23.34
Mean	1.331034	0.473	22.07655	22.99241	46.88862	1.191034	1.678276	25.18759
Stdev	0.398752	0.227321	2.464703	2.349743	3.025817	0.561461	0.428603	1.666966

MUFA	C - 18 : 2	C - 18 : 3	c-20:2	C - 20 : 4	C - 22:6	PUFA	C18:0/C18:	n3/n6
28.3	20.28	2.1	0.74	1.01	0.54	24.67	0.833967	3.876627
27.8	19.9	2.5	0.63	1.3	0.42	24.75	0.990244	4.451106
28.3	21.2	1.9	0.85	1.6	0.65	26.2	0.826087	4.38066
25.4	23.9	1.7	0.68	1.9	0.22	28.4	1.092827	4.289205
24.86	18.5	1.4	0.24	1.2	0.21	21.55	1.004484	2.851351
26.82	20.1	1.1	0.32	1.05	0.71	23.28	1.00823	2.505323
25.63	22.3	0.7	0.41	0.99	0.52	24.92	0.852614	2.123318
31.07	19.52	0.89	1.2	1.56	0.87	24.04	0.984012	3.69457
28.84	22.2	2	0.33	1.67	0.61	26.81	0.939567	4.027477
28.25	20.2	1.78	0.75	0.96	0.65	24.34	0.822171	3.522178
30.28	17.3	2.7	0.11	0.32	0.55	20.98	0.810646	3.161792
31.02	19.32	2.05	0.86	1.85	0.35	24.43	0.725275	4.778116
31.6	23.7	1.2	0.43	0.77	0.36	26.46	0.956264	2.41519
26.82	20.1	1.1	0.32	1.05	0.71	23.28	1.00823	2.505323
25.63	22.3	0.7	0.41	0.99	0.52	24.92	0.852614	2.123318
31.07	19.52	0.89	1.2	1.56	0.87	24.04	0.984012	3.69457
28.84	22.2	2	0.33	1.67	0.61	26.81	0.939567	4.027477
28.3	20.28	2.1	0.74	1.01	0.54	24.67	0.833967	3.876627
27.8	19.9	2.5	0.63	1.3	0.42	24.75	0.990244	4.451106
28.3	21.2	1.9	0.85	1.6	0.65	26.2	0.826087	4.38066
25.4	23.9	1.7	0.68	1.9	0.22	28.4	1.092827	4.289205
24.86	18.5	1.4	0.24	1.2	0.21	21.55	1.004484	2.851351
28.25	20.2	1.78	0.75	0.96	0.65	24.34	0.822171	3.522178
30.28	17.3	2.7	0.11	0.32	0.55	20.98	0.810646	3.161792
31.02	19.32	2.05	0.86	1.85	0.35	24.43	0.725275	4.778116
31.6	23.7	1.2	0.43	0.77	0.36	26.46	0.956264	2.41519
24.86	18.5	1.4	0.24	1.2	0.21	21.55	1.004484	2.851351
26.82	20.1	1.1	0.32	1.05	0.71	23.28	1.00823	2.505323
25.63	22.3	0.7	0.41	0.99	0.52	24.92	0.852614	2.123318
28.0569	20.61172	1.628966	0.554138	1.227586	0.508966	24.53138	0.915797	3.545649
2.231403	1.874456	0.601595	0.294843	0.428858	0.192119	1.991738	0.101842	0.869492

c18:2/C18:	C18:3/C18:1	sat/unsat
0.770517	0.079787234	26.33184
0.808943	0.101626016	26.44029
0.837945	0.075098814	27.90318
1.008439	0.071729958	30.25433
0.829596	0.062780269	23.31589
0.82716	0.04526749	25.04212
0.955441	0.029991431	26.71516
0.709302	0.032340116	25.74679
0.843786	0.076016724	28.422
0.777521	0.068514242	25.80832
0.657795	0.102661597	22.40602
0.707692	0.075091575	25.99061
0.878428	0.044477391	28.11253
0.82716	0.04526749	25.04212
0.955441	0.029991431	26.71516
0.709302	0.032340116	25.74679
0.843786	0.076016724	28.422
0.770517	0.079787234	26.33184
0.808943	0.101626016	26.44029
0.837945	0.075098814	27.90318
1.008439	0.071729958	30.25433
0.829596	0.062780269	23.31589
0.777521	0.068514242	25.80832
0.657795	0.102661597	22.40602
0.707692	0.075091575	25.99061
0.878428	0.044477391	28.11253
0.829596	0.062780269	23.31589
0.82716	0.04526749	25.04212
0.955441	0.029991431	26.71516
0.821977	0.074441548	26.20867
0.093747	0.022794942	2.029966
