# Supplementary material for: Radiotherapy improves serum fatty acids and lipid profile in breast cancer
Source: Lipids Health Dis. 2017 May 18;16:92. doi: 10.1186/s12944-017-0481-y (PMC5437547; doi:10.1186/s12944-017-0481-y)
Supplement: Supplementary file 5 — Serum lipid profile of pre treated BC patients. (PDF 162 kb) [file 12944_2017_481_MOESM5_ESM.pdf]

Control

	C - 14: 0	C-15:0	C - 16 : 0	C - 18 : 0	SFA	C 14:1	C - 16 : 1	C - 18 : 1
1	2.182139	0	12.6392	10.80181	25.62315	1.736203	2.367094	20.73493
2	1.440033	0	14.66196	19.31209	35.41409	0.269294	4.661961	20.04824
3	0	0.4	24.90226	18.25015	43.15242	0	0	16.12092
4	0	0.3	25.77806	10.02699	35.80505	0	3.303863	19.60403
5	2.484136	1.32	16.32606	14.36174	33.17194	0.229192	2.939904	18.74987
6	0	0	12.98722	9.98155	22.96877	0	3.3916	18.54336
7	1.512309	1.76	19.94444	14.53976	35.9965	0	1.675367	24.17404
8	0	0	24.90226	18.25015	43.15242	0	0	16.12092
9	0	1.9	25.77806	10.02699	35.80505	0	3.303863	19.60403
10	2.484136	2.8	16.32606	10.80181	25.62315	1.736203	2.367094	20.73493
11	2.182139	0	12.6392	19.31209	35.41409	0.269294	4.661961	20.04824
12	1.440033	0	14.66196	14.36174	33.17194	0.229192	2.939904	18.74987
13	0	0.4	12.98722	9.98155	22.96877	0	3.3916	18.54336
14	1.512309	0	19.94444	14.53976	35.9965	0	1.675367	24.17404
15	2.182139	2.3	12.6392	10.80181	25.62315	1.736203	2.367094	20.73493
16	1.440033	0	14.66196	19.31209	35.41409	0.269294	4.661961	20.04824
17	1.98765	2.5	12.98722	14.36174	33.17194	0.229192	2.939904	18.74987
18	1.512309	0	19.94444	9.98155	22.96877	0	3.3916	18.54336
19	0.866573	1.7	24.90226	14.53976	35.9965	0	1.675367	24.17404
20	0	1.23	25.77806	18.25015	43.15242	0	0	16.12092
21	2.484136	0	16.32606	10.02699	35.80505	0	3.303863	19.60403
22	0.9832	1.23	12.98722	14.36174	33.17194	0.229192	2.939904	18.74987
23	1.512309	0	19.94444	9.98155	22.96877	0	3.3916	18.54336
24	1.096	2.5	24.90226	14.53976	35.9965	0	1.675367	24.17404
25	0	0	25.77806	10.80181	25.62315	1.736203	2.367094	20.73493
26	2.484136	0	16.32606	19.31209	35.41409	0.269294	4.661961	20.04824
27	2.182139	0.3	12.6392	18.25015	43.15242	0	0	16.12092
28	1.440033	0.9	14.66196	10.02699	35.80505	0	3.303863	19.60403
29	1.5657	1.6	15.456	14.36174	33.17194	0.229192	2.939904	18.74987
30	0.005	1.1	11.9845	10.80181	25.62315	1.736203	2.367094	20.73493
Mean	1.23262	1.438125	18.24351	14.65483	33.16206	0.778868	2.631002	19.67764
Stdev	0.911304	0.83553	5.037451	3.562552	6.331743	0.740677	1.372192	2.283201

Total fatty acid

MUFA	C - 18 : 2	C - 18 : 3	C - 20 : 4	C - 20:2	C - 22:6	PUFA	C18:0/C18:	n3/n6
24.83823	32.8383	1.846906	11.79691	0.719876	0.123769	47.32576	0.520948	14.36746
24.97949	25.21181	0.178694	8.303023	2.100137	0	35.79366	0.963281	10.58185
16.12092	31.89112	0	5.415733	0	0	37.30686	1.132079	5.415733
22.9079	31.36122	0	7.3953	0	0	38.75652	0.511476	7.3953
21.91896	23.61601	1.411576	9.606536	2.75871	0.816649	38.20948	0.765965	13.8114
21.93496	30.77999	0	7.912082	6.119816	0	44.81189	0.538282	14.0319
25.84941	28.29885	0.16379	3.905618	0.208944	0.453679	33.03088	0.601462	4.294384
16.12092	31.89112	0	5.415733	0	0	37.30686	1.132079	5.415733
22.9079	31.36122	0	7.3953	0	0	38.75652	0.511476	7.3953
24.83823	32.8383	1.846906	11.79691	0.719876	0.123769	47.32576	0.520948	14.36746
24.97949	25.21181	0.178694	8.303023	2.100137	0	35.79366	0.963281	10.58185
21.91896	23.61601	1.411576	9.606536	2.75871	0.816649	38.20948	0.765965	13.8114
21.93496	30.77999	0	7.912082	6.119816	0	44.81189	0.538282	14.0319
25.84941	28.29885	0.16379	3.905618	0	0.453679	33.03088	0.601462	4.294384
24.83823	32.8383	1.846906	11.79691	0.719876	0.123769	47.32576	0.520948	14.36746
24.97949	25.21181	0.178694	8.303023	2.100137	0	35.79366	0.963281	10.58185
21.91896	23.61601	1.411576	9.606536	2.75871	0.816649	38.20948	0.765965	13.8114
21.93496	30.77999	0	7.912082	6.119816	0	44.81189	0.538282	14.0319
25.84941	28.29885	0.16379	3.905618	0.208944	0.453679	33.03088	0.601462	4.294384
16.12092	31.89112	0	5.415733	0	0	37.30686	1.132079	5.415733
22.9079	31.36122	0	7.3953	0	0	38.75652	0.511476	7.3953
21.91896	23.61601	1.411576	9.606536	2.75871	0.816649	38.20948	0.765965	13.8114
21.93496	30.77999	0	7.912082	0	0	44.81189	0.538282	14.0319
25.84941	28.29885	0.16379	3.905618	0.208944	0.453679	33.03088	0.601462	4.294384
24.83823	32.8383	1.846906	11.79691	0.719876	0.123769	47.32576	0.520948	5.367461
24.97949	25.21181	0.178694	8.303023	2.100137	0	35.79366	0.963281	10.58185
16.12092	31.89112	0	5.415733	0	0	37.30686	1.132079	5.415733
22.9079	31.36122	0	7.3953	0	0	38.75652	0.511476	7.3953
21.91896	23.61601	1.411576	9.606536	2.75871	0.816649	38.20948	0.765965	9.811402
24.83823	32.8383	1.846906	11.79691	0.719876	0.123769	47.32576	0.520948	14.36746
22.62477	28.9519	0.981241	7.72577	0.65	0.465486	39.28102	0.720687	8.323411
3.031294	3.281226	0.76397	2.429881	0.231909	0.303947	4.662034	0.227348	3.20123

c18:2/C18:	C18:3/C18:1	satu/unsat
1.583719	0.0890722	48.3573613
1.257557	0.0089132	37.2113867
1.978244	0	39.9836564
1.599733	0	40.3195201
1.259529	0.0752846	39.7228702
1.659893	0	45.8590207
1.17063	0.0067755	34.4234263
1.978244	0	39.9836564
1.599733	0	40.3195201
1.583719	0.0890722	48.3573613
1.257557	0.0089132	37.2113867
1.259529	0.0752846	39.7228702
1.659893	0	45.8590207
1.17063	0.0067755	34.4234263
1.583719	0.0890722	48.3573613
1.257557	0.0089132	37.2113867
1.259529	0.0752846	39.7228702
1.659893	0	45.8590207
1.17063	0.0067755	34.4234263
1.978244	0	39.9836564
1.599733	0	40.3195201
1.259529	0.0752846	39.7228702
1.659893	0	45.8590207
1.17063	0.0067755	34.4234263
1.583719	0.0890722	48.3573613
1.257557	0.0089132	37.2113867
1.978244	0	39.9836564
1.599733	0	40.3195201
1.259529	0.0752846	39.7228702
1.583719	0.0890722	48.3573613
1.492991	0.049141	41.0529733
0.270034	0.0383808	4.50765627
