# Supplementary material for: Radiotherapy improves serum fatty acids and lipid profile in breast cancer
Source: Lipids Health Dis. 2017 May 18;16:92. doi: 10.1186/s12944-017-0481-y (PMC5437547; doi:10.1186/s12944-017-0481-y)
Supplement: Supplementary file 6 — Serum lipid profile of post treated BC patients. (PDF 154 kb) [file 12944_2017_481_MOESM6_ESM.pdf]

Control Free

	C - 14: 0	C - 16 : 0	C - 18 : 0	SFA	C 14:1	C - 16 : 1	C - 18 : 1	MUFA
1	3.365197	22.1203	24.32838	49.81387	0.718751	3.373793	22.68861	26.78116
2	0.931092	18.44437	15.27828	34.65374	0.154608	3.46965	20.32159	23.94585
3	1.643152	10.08272	22.5569	34.28278	1.358811	4.52499	16.2702	22.154
4	1.864289	15.2141	23.23	40.30839	1.235647	3.155295	18.94653	23.33747
5	0.627388	18.26107	11.56826	30.45671	0.316065	2.526449	22.91771	25.76022
6	1.3417	23.61317	11.44516	36.40003	0	2.3417	20.11129	22.45299
7	1.287	24.52583	8.666017	34.47885	0	2.500597	20.55049	23.05109
8	1.643152	10.08272	22.5569	34.28278	1.358811	4.52499	16.2702	22.154
9	1.864289	15.2141	23.23	40.30839	1.235647	3.155295	18.94653	23.33747
10	0.627388	18.26107	11.56826	30.45671	0.316065	2.526449	22.91771	25.76022
11	3.365197	22.1203	24.32838	49.81387	0.718751	3.373793	22.68861	26.78116
12	0.931092	18.44437	15.27828	34.65374	0.154608	3.46965	20.32159	23.94585
13	0.627388	18.26107	11.56826	30.45671	0.316065	2.526449	22.91771	25.76022
14	1.3417	23.61317	11.44516	36.40003	0	2.3417	20.11129	22.45299
15	1.287	24.52583	8.666017	34.47885	0	2.500597	20.55049	23.05109
16	3.365197	22.1203	24.32838	49.81387	0.718751	3.373793	22.68861	26.78116
17	0.931092	18.44437	15.27828	34.65374	0.154608	3.46965	20.32159	23.94585
18	1.3417	23.61317	11.44516	36.40003	0	2.3417	20.11129	22.45299
19	1.287	24.52583	8.666017	34.47885	0	2.500597	20.55049	23.05109
20	1.643152	10.08272	12.5569	34.28278	1.358811	4.52499	16.2702	22.154
21	1.864289	15.2141	23.23	40.30839	1.235647	3.155295	18.94653	23.33747
22	0.627388	18.26107	11.56826	30.45671	0.316065	2.526449	22.91771	25.76022
23	1.3417	23.61317	11.44516	36.40003	0	2.3417	20.11129	22.45299
24	3.365197	22.1203	24.32838	49.81387	0.718751	3.373793	22.68861	26.78116
25	0.931092	18.44437	15.27828	34.65374	0.154608	3.46965	20.32159	23.94585
26	1.864289	15.2141	13.23	40.30839	1.235647	3.155295	18.94653	23.33747
27	1.287	24.52583	8.666017	34.47885	0	2.500597	20.55049	23.05109
28	1.643152	10.08272	152.5569	34.28278	1.358811	4.52499	16.2702	22.154
29	1.864289	15.2141	23.23	40.30839	1.235647	3.155295	18.94653	23.33747
30	0.627388	18.26107	11.56826	30.45671	0.316065	2.526449	12.91771	25.76022
Mean	1.267698	18.75071	13.89231	37.07809	0.756776	2.619839	19.713	23.967627
Stdev	0.929313	5.069059	3.561348	5.883396	0.492051	1.370378	2.213021	1.61257814

Fatty acid

C - 18 : 2	C - 18 : 3	C - 20 : 4	C-22:6	PUFA	C18:0/C18:	n3/n6	c18:2/C18:	C18:3/C18:1
11.65746	0.638643	5.033154	1.959019	19.28828	1.072273	5.839846	0.513802	0.02814818
26.76107	0	9.351494	0	36.11256	0.751825	9.351494	1.316879	0
22.30933	0	8.941002	0	31.25033	1.386394	8.941002	1.371177	0
28.75541	1.302986	4.790661	0	34.84906	1.226082	6.093647	1.517714	0.06877175
31.01399	2.512427	3.511335	0.444215	37.48197	0.504774	6.038085	1.353276	0.10962819
31.89112	0	5.415733	0	37.30686	0.569091	5.415733	1.585732	0
31.36122	0	7.3953	0	38.75652	0.421694	7.3953	1.526057	0
22.30933	0	8.941002	0	31.25033	1.386394	8.941002	1.371177	0
28.75541	1.302986	4.790661	0	34.84906	1.226082	6.093647	1.517714	0.06877175
31.01399	2.512427	3.511335	0.444215	37.48197	0.504774	6.038085	1.353276	0.10962819
11.65746	0.638643	5.033154	1.959019	19.28828	1.072273	5.839846	0.513802	0.02814818
26.76107	0	9.351494	0	36.11256	0.751825	9.351494	1.316879	0
31.01399	2.512427	3.511335	0.444215	37.48197	0.504774	6.038085	1.353276	0.10962819
31.89112	0	5.415733	0	37.30686	0.569091	5.415733	1.585732	0
31.36122	0	7.3953	0	38.75652	0.421694	7.3953	1.526057	0
11.65746	0.638643	5.033154	1.959019	19.28828	1.072273	5.839846	0.513802	0.02814818
26.76107	0	9.351494	0	36.11256	0.751825	9.351494	1.316879	0
31.89112	0	5.415733	0	37.30686	0.569091	5.415733	1.585732	0
31.36122	0	7.3953	0	38.75652	0.421694	7.3953	1.526057	0
22.30933	0	8.941002	0	31.25033	1.386394	8.941002	1.371177	0
28.75541	1.302986	4.790661	0	34.84906	1.226082	6.093647	1.517714	0.06877175
31.01399	2.512427	3.511335	0.444215	37.48197	0.504774	6.038085	1.353276	0.10962819
31.89112	0	5.415733	0	37.30686	0.569091	5.415733	1.585732	0
11.65746	0.638643	5.033154	1.959019	19.28828	1.072273	5.839846	0.513802	0.02814818
26.76107	0	9.351494	0	36.11256	0.751825	9.351494	1.316879	0
28.75541	1.302986	4.790661	0	34.84906	1.226082	6.093647	1.517714	0.06877175
31.36122	0	7.3953	0	38.75652	0.421694	7.3953	1.526057	0
22.30933	0	8.941002	0	31.25033	1.386394	8.941002	1.371177	0
28.75541	1.302986	4.790661	0	34.84906	1.226082	6.093647	1.517714	0.06877175
31.01399	2.512427	3.511335	0.444215	37.48197	0.504774	6.038085	1.353276	0.10962819
29.14923	1.545117	7.762189	1.117461	33.75045	0.719646	8.317739	1.520318	0.0717566
3.266907	0.797031	2.40299	0.798372	6.187945	0.249094	3.20085	0.285467	0.03376686

saturated/unsaturate
21.14831
37.55973
32.79781
36.57626
38.66429
38.92803
40.25228
32.79781
36.57626
38.66429
21.14831
37.55973
38.66429
38.92803
40.25228
21.14831
37.55973
38.92803
40.25228
32.79781
36.57626
38.66429
38.92803
21.14831
37.55973
36.57626
40.25228
32.79781
36.57626
38.66429
35.29824
6.051608
