# Supplementary material for: Radiotherapy improves serum fatty acids and lipid profile in breast cancer
Source: Lipids Health Dis. 2017 May 18;16:92. doi: 10.1186/s12944-017-0481-y (PMC5437547; doi:10.1186/s12944-017-0481-y)
Supplement: Supplementary file 7 — Serum lipid profile of controls. (PDF 232 kb) [file 12944_2017_481_MOESM7_ESM.pdf]

Pre Treated

No of samp	Age	TC	TG	HDL	LDL	VLDL	TC:HDL	HDL:LDL
1	34	253	148	52	185	29.6	4.86	0.28
2	38	203	139	40	145	27.8	5.07	0.296
3	42	252	140	43	178	28	5.86	0.24
4	36	226	163	47	156	32.6	4.8	0.3
5	45	198	169	39	133	33.8	5.07	0.29
6	47	232	154	48	142	30.8	4.83	0.33
7	45	211	171	91	138	34.2	2.31	0.65
8	30	268	160	79	188	32	3.39	0.42
9	33	216	167	56	139	33.4	3.85	0.4
10	25	241	107	72	174	21.4	3.34	0.41
11	52	249	142	83	176	28.4	3	0.47
12	40	191	179	42	128	35.8	7.95	0.18
13	56	152	129	60	112	25.8	2.53	0.53
14	60	230	168	54	140	33.6	4.25	0.38
15	62	231	180	46	141	36	5.02	0.32
16	43	204	113	42	138	22.6	4.85	0.3
17	36	253	124	83	183	24.8	3.04	0.45
18	39	270	121	34	188	24.2	7.94	0.18
19	40	239	115	28	153	23	8.53	0.18
20	46	235	142	39	142	28.4	6.02	0.27
21	47	216	137	44	118	27.4	4.9	0.37
22	33	198	126	46	105	25.2	4.3	0.43
23	46	205	164	75	112	32.8	2.73	0.66
24	38	249	154	62	136	30.8	4.01	0.45
25	33	204	163	73	112	32.6	2.79	0.65
26	60	210	128	84	111	25.6	2.5	0.75
27	58	225	132	55	126	26.4	4.09	0.43
28	46	213	144	59	128	28.8	3.61	0.46
29	57	228	156	61	130	31.2	3.73	0.46
30	63	230	108	42	132	21.6	5.47	0.31
31	33	249	142	83	118	28.4	3	0.47
32	25	191	179	42	105	35.8	7.95	0.18
33	52	152	129	60	112	25.8	2.53	0.53
34	40	230	168	54	136	33.6	4.25	0.38
35	56	231	180	46	112	36	5.02	0.32
36	60	204	113	42	111	22.6	4.85	0.3
37	62	253	124	83	126	24.8	3.04	0.45
38	43	270	121	34	128	24.2	7.94	0.18
39	36	239	115	28	130	23	8.53	0.18
40	39	235	142	52	132	29.6	4.86	0.28
41	40	253	148	40	176	27.8	5.07	0.296
42	46	203	139	43	128	28	5.86	0.24
43	47	252	140	47	112	32.6	4.8	0.3
44	33	226	163	39	140	33.8	5.07	0.29
45	46	198	169	48	141	30.8	4.83	0.33
46	34	232	154	91	138	34.2	2.31	0.65

47	38	211	171	79	183	32	3.39	0.42
48	42	268	160	56	188	33.4	3.85	0.4
49	36	216	167	72	153	21.4	3.34	0.41
50	45	241	107	39	142	27.4	4.9	0.37
51	47	216	137	44	185	25.2	4.3	0.43
52	45	198	126	46	145	32.8	2.73	0.66
53	30	205	164	75	178	30.8	4.01	0.45
54	38	249	154	62	156	32.6	2.79	0.65
55	33	204	163	73	133	25.6	2.5	0.75
56	60	210	128	84	142	26.4	4.09	0.43
57	58	225	132	55	138	28.8	3.61	0.46
58	46	213	144	59	188	31.2	3.73	0.46
59	57	228	156	61	139	21.6	5.47	0.31
60	63	230	108	42	174	29.6	4.86	0.28
61	33	253	148	52	176	27.8	5.07	0.296
62	25	203	139	40	128	28	5.86	0.24
63	52	252	140	43	112	32.6	4.8	0.3
64	40	226	163	47	140	33.8	5.07	0.29
65	56	198	169	39	141	30.8	4.83	0.33
66	33	232	154	48	138	34.2	2.31	0.65
67	25	211	171	91	183	32	3.39	0.42
68	52	268	160	79	188	33.4	3.85	0.4
69	40	216	167	56	153	21.4	3.34	0.41
70	56	241	107	72	142	27.4	4.9	0.37
71	60	249	142	44	185	25.2	4.3	0.43
72	62	191	179	46	145	32.8	2.73	0.66
73	43	152	129	75	178	30.8	4.01	0.45
74	36	230	168	62	156	32.6	2.79	0.65
75	39	231	180	73	133	25.6	2.5	0.75
76	34	204	113	84	142	26.4	4.09	0.43
77	38	253	124	55	138	28.8	3.61	0.46
78	42	270	121	59	188	31.2	3.73	0.46
79	36	239	115	61	139	21.6	5.47	0.31
80	45	235	142	42	174	28.4	3	0.47
81	47	216	137	83	118	28.4	3	0.47
82	45	198	126	42	105	35.8	7.95	0.18
83	30	205	164	60	112	25.8	2.53	0.53
84	33	249	154	54	136	33.6	4.25	0.38
85	25	204	163	46	112	36	5.02	0.32
86	52	210	128	42	111	22.6	4.85	0.3
87	43	225	132	83	126	24.8	3.04	0.45
88	54	213	144	34	128	24.2	7.94	0.18
89	29	228	156	28	130	23	8.53	0.18
90	34	230	108	39	132	27.4	4.9	0.37
91	33	216	142	44	185	25.2	4.3	0.43
92	25	198	179	46	145	32.8	2.73	0.66
93	52	205	129	75	178	30.8	4.01	0.45
94	40	249	168	62	156	32.6	2.79	0.65
95	56	204	180	73	133	25.6	2.5	0.75
96	60	210	113	84	142	26.4	4.09	0.43

97	62	225	124	55	138	28.8	3.61	0.46
98	43	213	121	59	188	31.2	3.73	0.46
99	36	228	115	61	139	21.6	5.47	0.31
100	39	230	142	42	174	28.4	3	0.47
101	40	249	137	83	118	35.8	7.95	0.18
102	46	191	126	42	105	25.8	2.53	0.53
103	47	152	164	60	112	33.6	4.25	0.38
104	33	230	154	54	136	36	5.02	0.32
105	46	231	163	46	112	22.6	4.85	0.3
106	38	204	128	42	111	24.8	3.04	0.45
107	33	253	132	83	126	24.2	7.94	0.18
108	60	270	144	34	128	23	8.53	0.18
109	58	239	156	28	130	28.4	6.02	0.27
110	46	235	108	39	132	29.6	4.86	0.28
111	57	253	148	52	176	27.8	5.07	0.296
112	63	203	139	40	128	28	5.86	0.24
113	33	252	140	43	112	32.6	4.8	0.3
114	34	226	163	47	140	33.8	5.07	0.29
115	38	198	169	39	141	30.8	4.83	0.33
116	42	232	154	48	138	34.2	2.31	0.65
117	36	211	171	91	183	32	3.39	0.42
118	45	268	160	79	188	33.4	3.85	0.4
119	47	216	167	56	153	21.4	3.34	0.41
120	45	241	107	72	142	28.4	6.02	0.27
121	30	231	142	39	142	27.4	4.9	0.37
122	33	204	137	44	118	25.2	4.3	0.43
123	25	253	126	46	105	32.8	2.73	0.46
124	52	270	164	75	112	30.8	4.01	0.45
125	34	239	154	77	136	32.6	2.79	0.5
126	38	235	176	73	112	25.6	2.5	0.55
127	42	216	128	84	111	26.4	4.09	0.43
128	36	198	132	76	126	28.8	3.61	0.46
129	45	205	144	75	128	31.2	3.73	0.36
130	56	249	156	61	130	21.6	5.47	0.31
Mean		224.8308	144.8538	56.66154	141.3538	28.89846	4.412846	0.399262
Stvd		24.77625	20.64732	16.83599	24.6186	4.155374	1.551258	0.139517

TC:LDL	TL
1.367568	788
1.4	677
1.41573	763
1.448718	742
1.488722	689
1.633803	726
1.528986	761
1.425532	845
1.553957	728
1.385057	744
1.414773	800
1.492188	690
1.357143	603
1.642857	742
1.638298	748
1.478261	647
1.382514	793
1.43617	763
1.562092	685
1.65493	708
1.830508	665
1.885714	625
1.830357	706
1.830882	751
1.821429	702
1.891892	683
1.785714	688
1.664063	694
1.753846	725
1.742424	662
1.414773	800
1.492188	690
1.357143	603
1.642857	742
1.638298	748
1.478261	647
1.382514	793
1.43617	763
1.562092	685
1.367568	788
1.4	677
1.41573	763
1.448718	742
1.488722	689
1.633803	726
1.528986	761

1.425532	845
1.553957	728
1.385057	744
1.830508	665
1.885714	625
1.830357	706
1.830882	751
1.821429	702
1.891892	683
1.785714	688
1.664063	694
1.753846	725
1.742424	662
1.367568	788
1.4	677
1.41573	763
1.448718	742
1.488722	689
1.633803	726
1.528986	761
1.425532	845
1.553957	728
1.385057	744
1.830508	665
1.885714	625
1.830357	706
1.830882	751
1.821429	702
1.891892	683
1.785714	688
1.664063	694
1.753846	725
1.742424	662
1.414773	800
1.414773	800
1.492188	690
1.357143	603
1.642857	742
1.638298	748
1.478261	647
1.382514	793
1.43617	763
1.562092	685
1.830508	665
1.885714	625
1.830357	706
1.830882	751
1.821429	702
1.891892	683
1.785714	688

1.664063	694
1.753846	725
1.742424	662
1.414773	800
1.492188	690
1.357143	603
1.642857	742
1.638298	748
1.478261	647
1.382514	793
1.43617	763
1.562092	685
1.65493	708
1.367568	788
1.4	677
1.41573	763
1.448718	742
1.488722	689
1.633803	726
1.528986	761
1.425532	845
1.553957	728
1.385057	744
1.65493	708
1.730508	665
1.885714	625
1.630357	706
1.530882	751
1.821429	702
1.791892	763
1.785714	688
1.664063	734
1.653846	725
1.742424	662
1.599794	717.5769
0.174192	52.89786
