# Supplementary material for: Radiotherapy improves serum fatty acids and lipid profile in breast cancer
Source: Lipids Health Dis. 2017 May 18;16:92. doi: 10.1186/s12944-017-0481-y (PMC5437547; doi:10.1186/s12944-017-0481-y)
Supplement: Supplementary file 8 — GC-FID Chromatogram showing serum total fatty acid profile of pre-treated BC patients. (PDF 233 kb) [file 12944_2017_481_MOESM8_ESM.pdf]

Post

No Of Sample	Age	TC	TG	HDL	LDL	VLDL	TC:LDl	TC:HDL
1	34	122	130	43	70	26	1.742857	2.83
2	38	142	169	39	79	33.8	1.797468	3.64
3	42	158	228	41	118	45.6	1.338983	3.85
4	36	126	207	44	86	41.4	1.465116	2.86
5	45	120	123	38	67	24.6	1.791045	3.15
6	47	189	178	49	127	35.6	1.488189	3.85
7	45	152	145	45	96	38.2	1.583333	3.37
8	30	158	157	37	85	31.4	1.858824	4.27
9	33	139	143	40	85	44.2	1.635294	3.47
10	25	169	151	35	76	30.2	2.223684	4.82
11	52	121	77	47	49	35.4	2.469388	2.57
12	40	103	157	43	58	31.4	1.775862	2.39
13	56	136	133	52	84	26.6	1.619048	2.61
14	60	174	114	50	112	22.8	1.553571	3.48
15	62	195	128	53	159	36.4	1.226415	3.67
16	43	169	132	44	148	26.4	1.141892	3.84
17	36	143	126	43	76	25.2	1.881579	3.32
18	39	135	160	54	89	32	1.516854	2.5
19	40	144	136	36	102	27.2	1.411765	4
20	46	152	125	42	112	25	1.357143	3.61
21	47	164	143	39	62	28.4	2.645161	4.2
22	33	180	134	32	57	30.6	3.157895	5.62
23	46	170	142	43	84	35	2.02381	3.95
24	38	156	162	47	86	33.6	1.813953	3.95
25	33	167	168	41	123	33.8	1.357724	4.07
26	60	123	135	38	87	27	1.413793	3.23
27	58	168	148	40	48	29.6	3.5	4.2
28	46	147	142	45	98	28.4	1.5	3.26
29	57	155	141	50	93	28.2	1.666667	3.1
30	63	130	154	51	79	30.8	1.64557	2.54
31	33	121	77	47	70	26	1.742857	2.83
32	25	103	157	43	79	33.8	1.797468	3.64
33	52	136	133	52	118	45.6	1.338983	3.85
34	40	174	114	50	86	41.4	1.465116	2.86
35	56	195	128	53	67	24.6	1.791045	3.15
36	60	169	132	44	127	35.6	1.488189	3.85
37	62	143	126	43	96	38.2	1.583333	3.37
38	43	135	160	54	85	31.4	1.858824	4.27
39	36	144	136	36	85	44.2	1.635294	3.47
40	39	152	125	42	76	30.2	2.223684	4.82
41	40	122	130	43	62	28.4	2.645161	4.2
42	46	142	169	39	57	30.6	3.157895	5.62
43	47	158	228	41	84	35	2.02381	3.95
44	33	126	207	44	86	33.6	1.813953	3.95
45	46	120	123	38	123	33.8	1.357724	4.07
46	34	189	178	49	87	27	1.413793	3.23
47	38	152	145	45	48	29.6	3.5	4.2

48	42	158	157	37	98	28.4	1.5	3.26
49	36	139	143	40	93	28.2	1.666667	3.1
50	45	169	151	35	79	30.8	1.64557	2.54
51	47	164	143	39	49	35.4	2.469388	2.57
52	45	180	134	32	58	31.4	1.775862	2.39
53	30	170	142	43	84	26.6	1.619048	2.61
54	38	156	162	47	112	22.8	1.553571	3.48
55	33	167	168	41	159	36.4	1.226415	3.67
56	60	123	135	38	148	26.4	1.141892	3.84
57	58	168	148	40	76	25.2	1.881579	3.32
58	46	147	142	45	89	32	1.516854	2.5
59	57	155	141	50	102	27.2	1.411765	4
60	63	130	154	51	112	25	1.357143	3.61
61	33	122	130	43	62	28.4	2.645161	4.2
62	25	142	169	39	57	30.6	3.157895	5.62
63	52	158	228	41	84	35	2.02381	3.95
64	40	126	207	44	86	33.6	1.813953	3.95
65	56	120	123	38	123	33.8	1.357724	4.07
66	33	189	178	49	87	27	1.413793	3.23
67	25	152	145	45	48	29.6	3.5	4.2
68	52	158	157	37	98	28.4	1.5	3.26
69	40	139	143	40	93	28.2	1.666667	3.1
70	56	169	151	35	79	30.8	1.64557	2.54
71	60	164	143	39	49	35.4	2.469388	2.57
72	62	180	134	32	58	31.4	1.775862	2.39
73	43	170	142	43	84	26.6	1.619048	2.61
74	36	156	162	47	112	22.8	1.553571	3.48
75	39	167	168	41	159	36.4	1.226415	3.67
76	34	123	135	38	148	26.4	1.141892	3.84
77	38	168	148	40	76	25.2	1.881579	3.32
78	42	147	142	45	89	32	1.516854	2.5
79	36	155	141	50	102	27.2	1.411765	4
80	45	130	154	51	112	25	1.357143	3.61
81	47	121	77	47	70	26	1.742857	2.83
82	45	103	157	43	79	33.8	1.797468	3.64
83	30	136	133	52	118	45.6	1.338983	3.85
84	33	174	114	50	86	41.4	1.465116	2.86
85	25	195	128	53	67	24.6	1.791045	3.15
86	52	169	132	44	127	35.6	1.488189	3.85
87	43	143	126	43	96	38.2	1.583333	3.37
88	54	135	160	54	85	31.4	1.858824	4.27
89	29	144	136	36	85	44.2	1.635294	3.47
90	34	152	125	42	76	30.2	2.223684	4.82
91	33	164	143	39	49	35.4	2.469388	2.57
92	25	180	134	32	58	31.4	1.775862	2.39
93	52	170	142	43	84	26.6	1.619048	2.61
94	40	156	162	47	112	22.8	1.553571	3.48
95	56	167	168	41	159	36.4	1.226415	3.67
96	60	123	135	38	148	26.4	1.141892	3.84
97	62	168	148	40	76	25.2	1.881579	3.32

98	43	147	142	45	89	32	1.516854	2.5	
99	36	155	141	50	102	27.2	1.411765	4	
100	39	130	154	51	112	25	1.357143	3.61	
101	40	121	77	47	70	26	1.742857	2.83	
102	46	103	157	43	79	33.8	1.797468	3.64	
103	47	136	133	52	118	45.6	1.338983	3.85	
104	33	174	114	50	86	41.4	1.465116	2.86	
105	46	195	128	53	67	24.6	1.791045	3.15	
106	38	169	132	44	127	35.6	1.488189	3.85	
107	33	143	126	43	96	38.2	1.583333	3.37	
108	60	135	160	54	85	31.4	1.858824	4.27	
109	58	144	136	36	85	44.2	1.635294	3.47	
110	46	152	125	42	76	30.2	2.223684	4.82	
111	57	122	130	43	62	28.4	2.645161	4.2	
112	63	142	169	39	57	30.6	3.157895	5.62	
113	33	158	228	41	84	35	2.02381	3.95	
114	34	126	207	44	86	33.6	1.813953	3.95	
115	38	120	123	38	123	33.8	1.357724	4.07	
116	42	189	178	49	87	27	1.413793	3.23	
117	36	152	145	45	48	29.6	3.5	4.2	
118	45	158	157	37	98	28.4	1.5	3.26	
119	47	139	143	40	93	28.2	1.666667	3.1	
120	45	169	151	35	79	30.8	1.64557	2.54	
121	30	152	125	42	148	26.4	1.141892	3.84	
122	33	164	143	39	76	25.2	1.881579	3.32	
123	25	180	134	32	89	32	1.516854	2.5	
124	52	170	142	43	102	27.2	1.411765	4	
125	34	156	162	47	112	25	1.357143	3.61	
126	38	167	168	41	62	28.4	2.645161	4.2	
127	42	123	135	38	57	30.6	3.157895	5.62	
128	36	168	148	40	84	35	2.02381	3.95	
129	45	147	142	45	86	33.6	1.813953	3.95	
130	56	155	141	50	123	33.8	1.357724	4.07	
Mean		150.2333	146.2667	43.36667	89.83333	31.49333	1.786763	3.540667	
Stdev		22.19612	27.42253	5.623125	26.6886	5.715975	0.53787	0.726209	

HDL:LDL	TL
0.61	515
0.49	579
0.34	695
0.51	613
0.56	498
0.38	693
0.46	588
0.45	587
0.47	557
0.46	581
0.95	444
0.74	511
0.61	555
0.44	600
0.33	685
0.29	643
0.56	538
0.6	588
0.35	568
0.37	581
0.62	558
0.56	553
0.51	589
0.54	601
0.33	649
0.43	533
0.83	554
0.45	582
0.53	589
0.64	564
0.61	515
0.49	579
0.34	695
0.51	613
0.56	498
0.38	693
0.46	588
0.45	587
0.47	557
0.46	581
0.62	558
0.56	553
0.51	589
0.54	601
0.33	649
0.43	533
0.83	554

0.45	582
0.53	589
0.64	564
0.95	444
0.74	511
0.61	555
0.44	600
0.33	685
0.29	643
0.56	538
0.6	588
0.35	568
0.37	581
0.62	558
0.56	553
0.51	589
0.54	601
0.33	649
0.43	533
0.83	554
0.45	582
0.53	589
0.64	564
0.95	444
0.74	511
0.61	555
0.44	600
0.33	685
0.29	643
0.56	538
0.6	588
0.35	568
0.37	581
0.61	515
0.49	579
0.34	695
0.51	613
0.56	498
0.38	693
0.46	588
0.45	587
0.47	557
0.46	581
0.95	444
0.74	511
0.61	555
0.44	600
0.33	685
0.29	643
0.56	538

0.6	588
0.35	568
0.37	581
0.61	515
0.49	579
0.34	695
0.51	613
0.56	498
0.38	693
0.46	588
0.45	587
0.47	557
0.46	581
0.62	558
0.56	553
0.51	589
0.54	601
0.33	649
0.43	533
0.83	554
0.45	582
0.53	589
0.64	564
0.29	643
0.56	538
0.6	588
0.35	568
0.37	581
0.62	558
0.56	553
0.51	589
0.54	601
0.33	649
0.513667	579.7
0.149031	55.59841
