# Supplementary material for: Radiotherapy improves serum fatty acids and lipid profile in breast cancer
Source: Lipids Health Dis. 2017 May 18;16:92. doi: 10.1186/s12944-017-0481-y (PMC5437547; doi:10.1186/s12944-017-0481-y)
Supplement: Supplementary file 9 — Serum free fatty acids in post-treated BC patients. (PDF 159 kb) [file 12944_2017_481_MOESM9_ESM.pdf]

Control

No Of Sam|	Age	TC	TG	HDL	LDL	VLDL	TC:HDL	HDL:LDL
1	35	175	114	40	38	22.8	4.375	1.052632
2	38	154	94	60	81	18.8	2.566667	0.740741
3	25	141	93	56	31	18.6	2.517857	1.806452
4	38	158	88	39	57	17.6	4.051282	0.684211
5	30	135	99	47	41	19.8	2.87234	1.146341
6	30	110	54	52	72	10.8	2.115385	0.722222
7	60	90	113	63	36	22.6	1.428571	1.75
8	52	138	105	46	57	21	3	0.807018
9	35	121	142	56	42	28.4	2.160714	1.333333
10	32	129	74	52	23	14.8	2.480769	2.26087
11	33	89	130	46	34	26	1.934783	1.352941
12	35	128	149	69	44	29.8	1.855072	1.568182
13	28	92	96	51	46	19.2	1.803922	1.108696
14	38	127	95	61	70	19	2.081967	0.871429
15	35	153	181	67	48	36.2	2.283582	1.395833
16	33	122	57	44	58	11.4	2.772727	0.758621
17	40	167	113	51	58	22.6	3.27451	0.87931
18	25	142	112	49	49	22.4	2.897959	1
19	38	178	163	72	57	20.6	2.472222	1.263158
20	45	146	91	50	51	18.2	2.92	0.980392
21	31	156	119	58	54	23.8	2.689655	1.074074
22	40	171	153	60	58	30.6	2.85	1.034483
23	63	115	112	47	48	24.4	2.446809	0.979167
24	47	192	120	56	51	24	3.428571	1.098039
25	25	116	116	44	52	23.2	2.636364	0.846154
26	28	150	118	65	62	24.4	2.307692	1.048387
27	25	125	126	55	42	23.6	2.272727	1.309524
28	26	105	141	63	79	25.2	1.666667	0.797468
29	32	122	132	52	74	28.2	2.346154	0.702703
30	26	113	105	54	63	26.4	2.092593	0.857143
31	63	115	99	42	57	21	2.738095	0.736842
32	42	168	91	60	52	19.8	3.8	1.153846
33	47	126	102	63	62	18.2	2.9	1.016129
34	56	139	126	60	57	20.4	2.316667	1.052632
35	54	142	103	65	43	25.2	3.184615	1.511628
36	47	113	96	69	55	20.6	3.637681	1.254545
37	49	139	126	48	70	19.2	2.895833	0.685714
38	58	147	109	59	68	25.2	2.491525	0.867647
39	56	152	105	47	62	21.8	3.234043	0.758065
40	36	113	136	65	56	21	3.738462	1.160714
41	28	149	129	68	50	27.2	2.191176	1.079365
42	48	156	92	58	64	25.8	2.689655	0.90625
43	42	104	140	63	64	18.4	3.650794	0.984375
44	37	151	118	64	57	28	2.359375	0.222807
45	62	142	86	61	53	23.6	2.327869	1.150943
46	34	133	123	46	60	17.2	3.891304	0.766667

	47	47	145	146	72	57	24.6	2.013889	1.074627
	48	46	185	140	70	69	29.2	3.642857	1.214493
	49	60	173	125	67	55	28	2.58209	1.218182
	50	51	142	124	56	52	24.8	3.535714	1.016923
Mean			137.88	114.42	56.56	54.78	22.672	2.728484	1.061238
Stdev			24.4504	24.71477	14.77	11.9203	4.750211	0.84	0.453

TC:LDL	TL
4.605263	517
1.901235	539
4.548387	471
2.77193	492
3.292683	472
1.527778	438
2.5	452
2.421053	496
2.880952	511
5.608696	428
2.617647	449
2.909091	540
2	435
1.814286	503
3.1875	599
2.103448	431
2.87931	539
2.897959	502
3.122807	620
2.862745	488
2.888889	537
2.948276	592
2.395833	472
3.764706	569
2.230769	478
2.419355	545
2.97619	498
1.329114	538
1.648649	530
1.793651	485
2.017544	463
3.230769	521
2.032258	503
2.438596	432
3.302326	503
2.054545	483
3.985714	533
2.161765	533
2.451613	516
2.017857	520
2.365079	559
2.4375	420
3.625	521
2.649123	540
2.679245	499
2.216667	552

2.164179	480
2.681159	614
3.145455	570
2.730769	524
2.704707	509.04
0.808072	14.7456
