# Supplementary material for: Radiotherapy improves serum fatty acids and lipid profile in breast cancer
Source: Lipids Health Dis. 2017 May 18;16:92. doi: 10.1186/s12944-017-0481-y (PMC5437547; doi:10.1186/s12944-017-0481-y)
Supplement: Supplementary file 10 — Serum total fatty acids in post treated BC patients. (PDF 402 kb) [file 12944_2017_481_MOESM10_ESM.pdf]

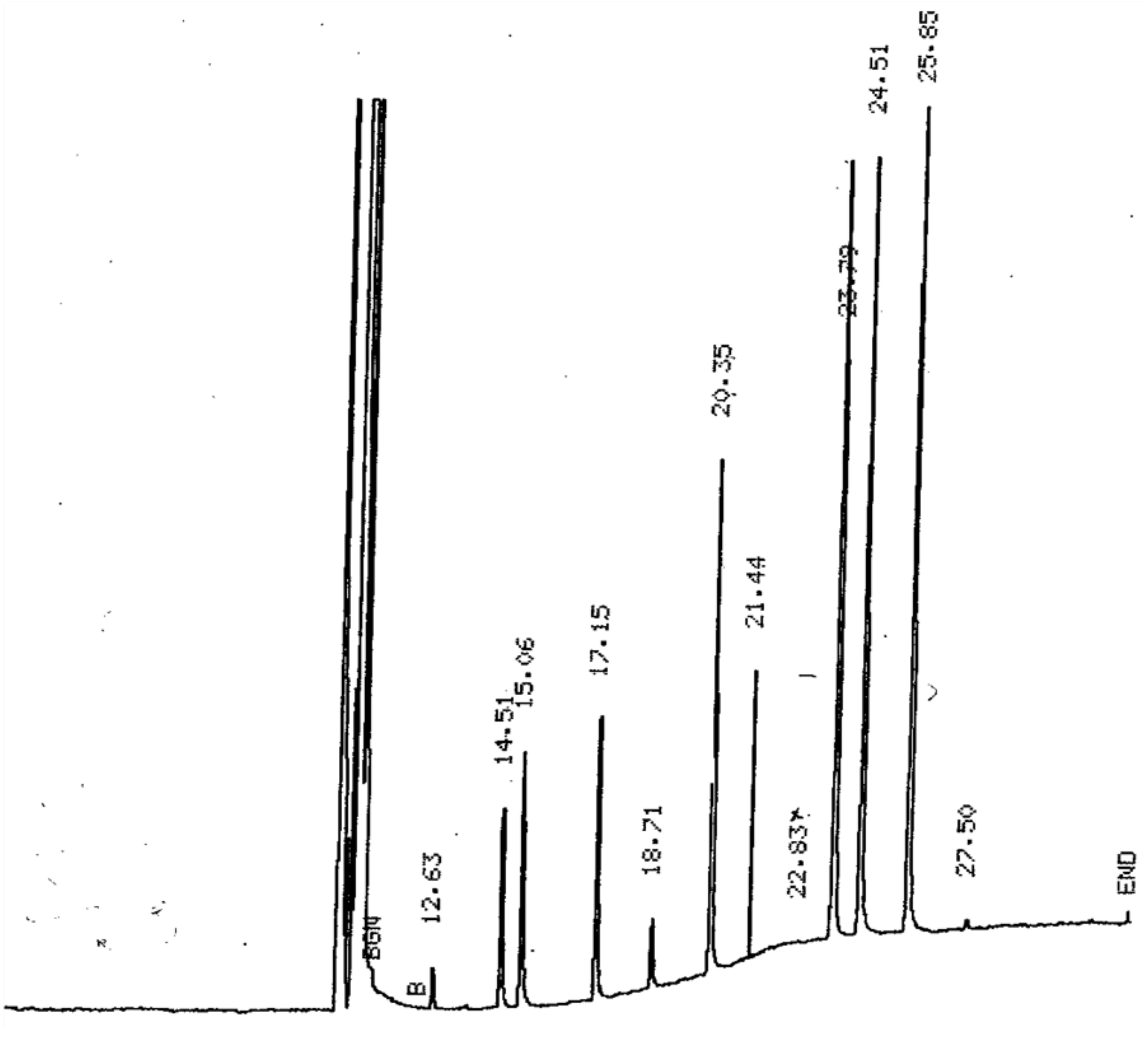

GC-FID chromatogram showing total serum fatty acid profile of Pre- treated BC patients

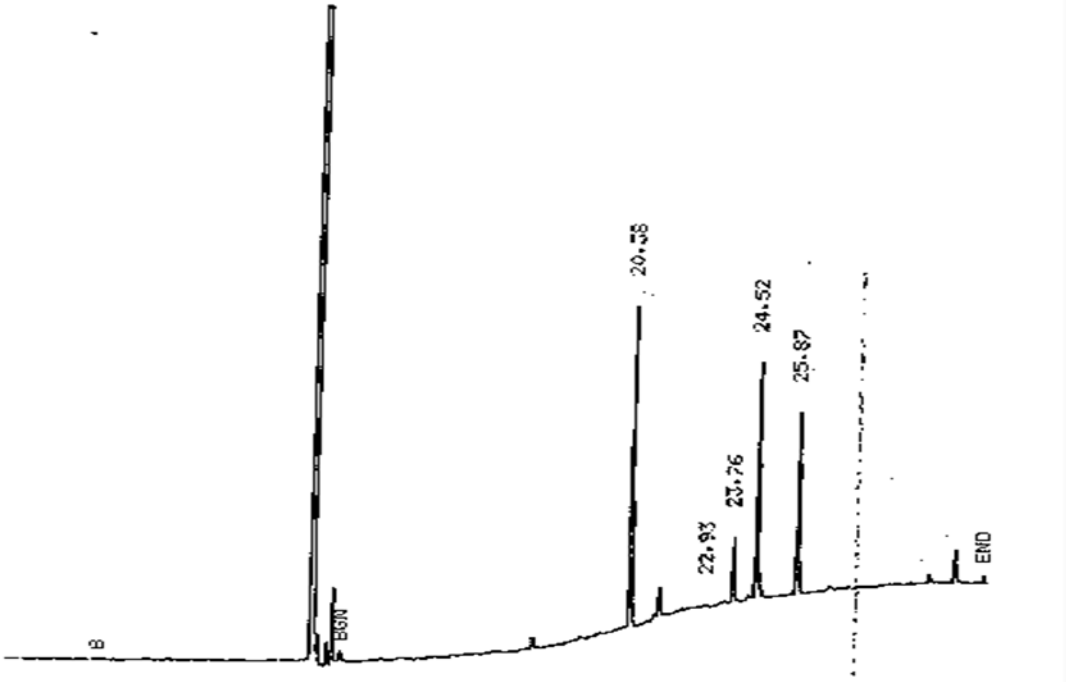

GC-FID chromatogram showing free serum fatty acid profile of Pre- treated BC patients

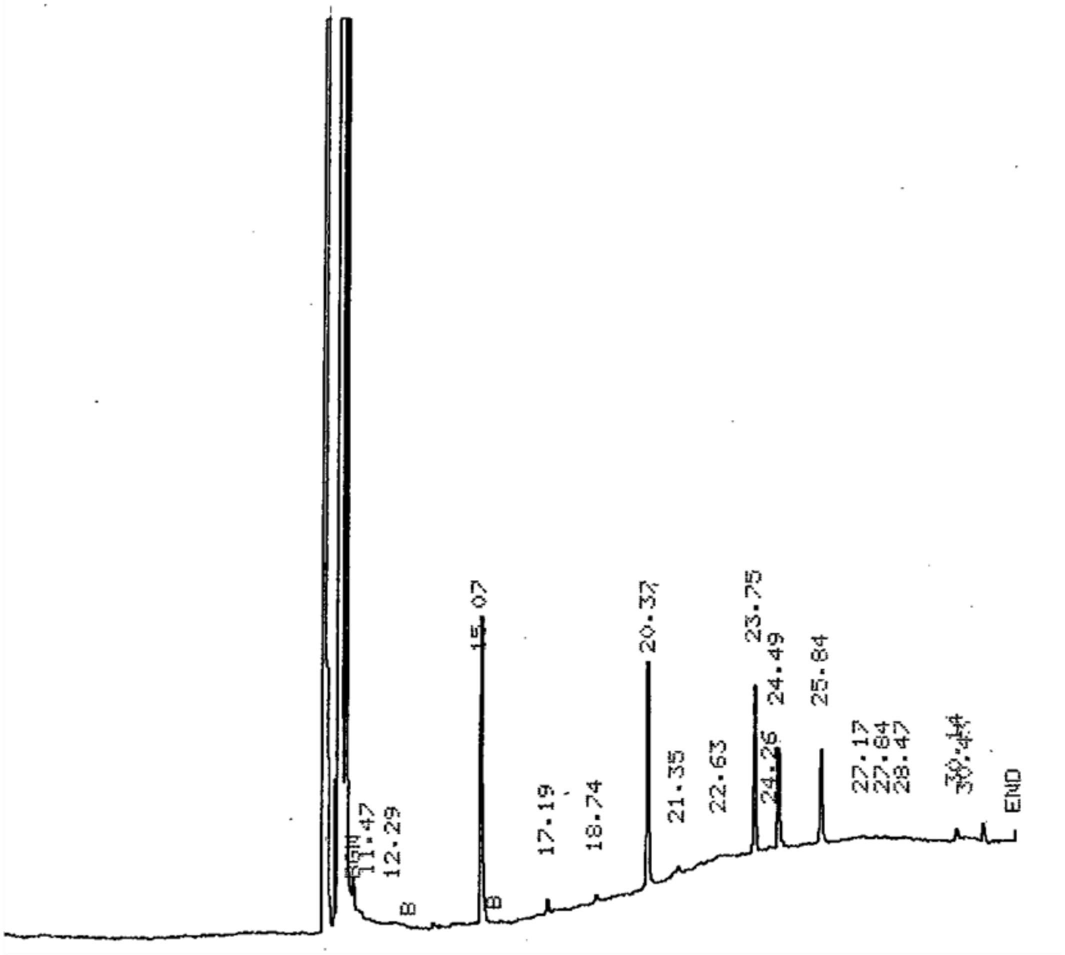

GC-FID chromatogram showing total serum fatty acid profile of Post treated BC patients

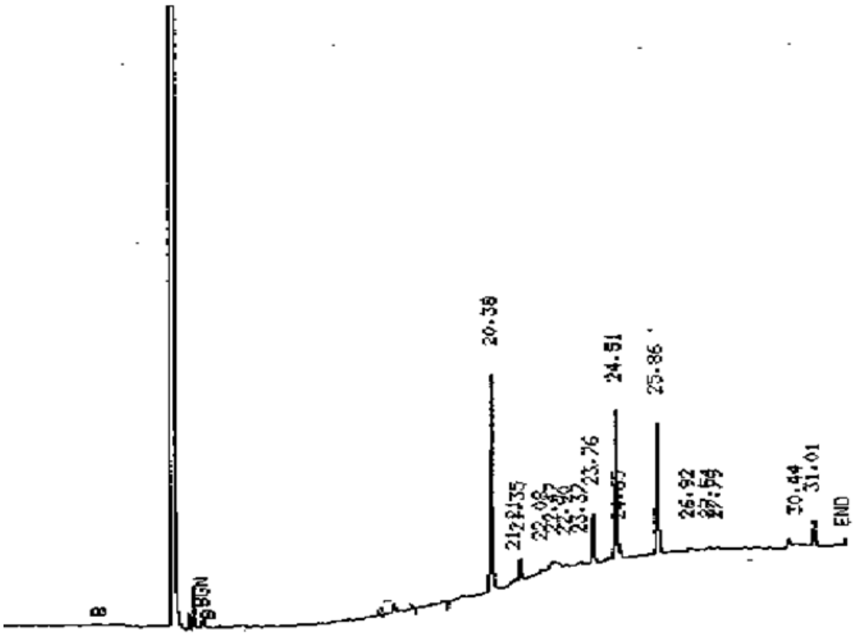

GC-FID chromatogram showing free serum fatty acid profile of Post-treated BC patients

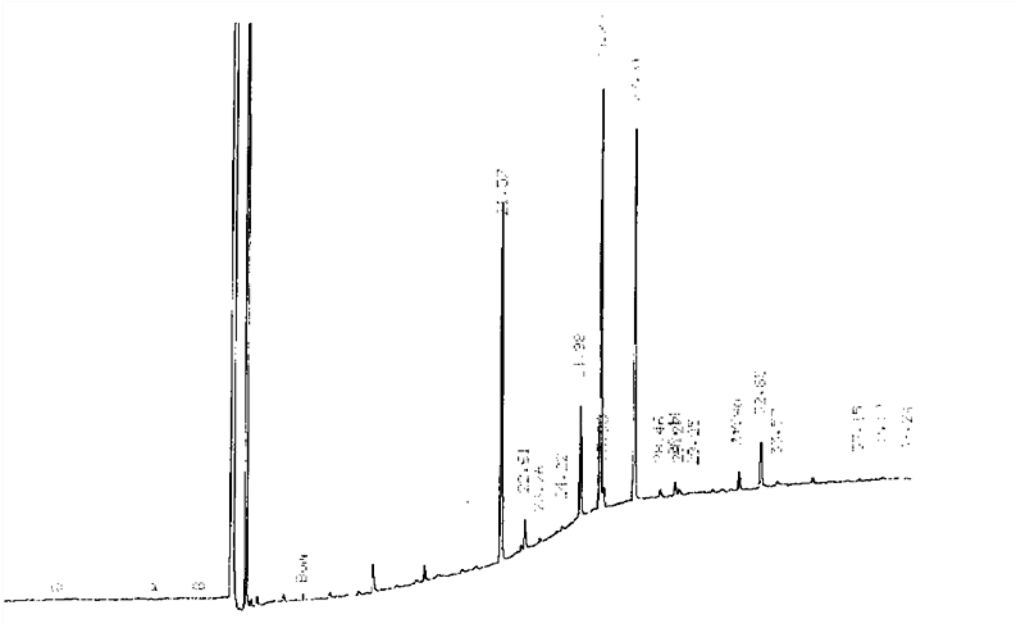

GC-FID chromatogram showing free serum fatty acid profile of control

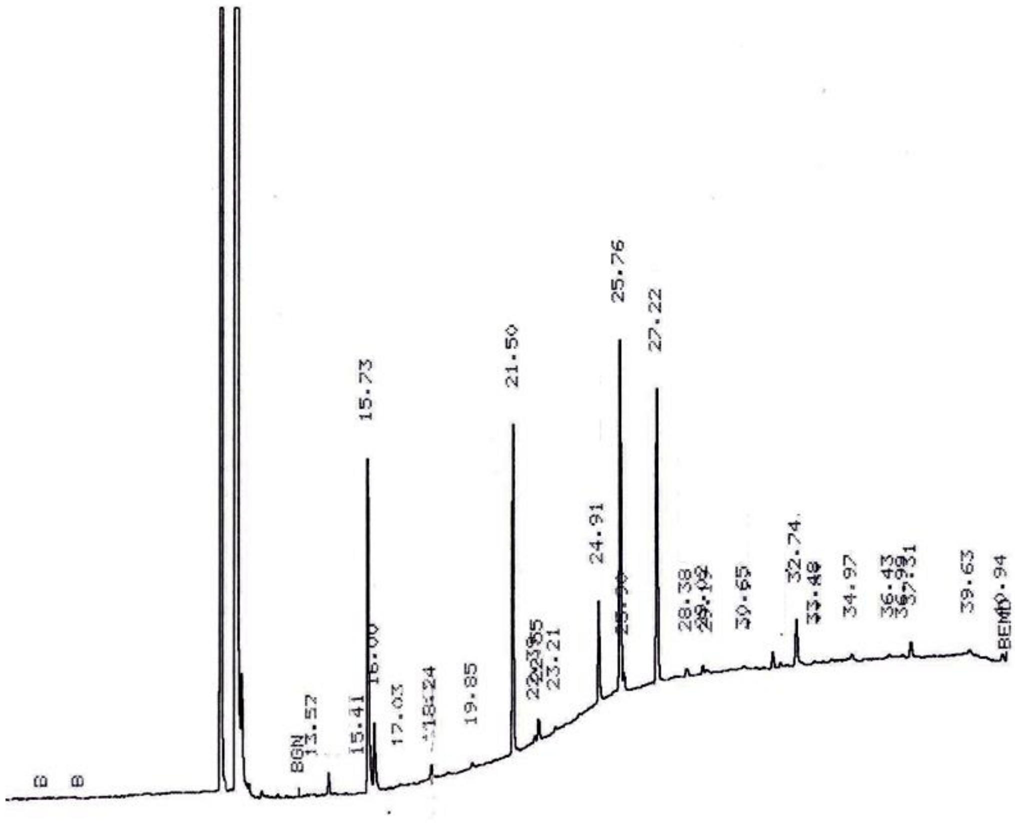

GC-FID chromatogram showing total serum fatty acid profile of control
